# Supramolecular Organization of Nonstoichiometric Drug Hydrates: Dapsone

**DOI:** 10.3389/fchem.2018.00031

**Published:** 2018-02-22

**Authors:** Doris E. Braun, Ulrich J. Griesser

**Affiliations:** Institute of Pharmacy, University of Innsbruck, Innsbruck, Austria

**Keywords:** dapsone, hydrate, crystal structure prediction, temperature and moisture dependent stability, intermolecular energy

## Abstract

The observed moisture- and temperature dependent transformations of the dapsone (4,4′-diaminodiphenyl sulfone, DDS) 0. 33-hydrate were correlated to its structure and the number and strength of the water-DDS intermolecular interactions. A combination of characterization techniques was used, including thermal analysis (hot-stage microscopy, differential scanning calorimetry and thermogravimetric analysis), gravimetric moisture sorption/desorption studies and variable humidity powder X-ray diffraction, along with computational modeling (crystal structure prediction and pair-wise intermolecular energy calculations). Depending on the relative humidity the hydrate contains between 0 and 0.33 molecules of water per molecule DDS. The crystal structure is retained upon dehydration indicating that DDS hydrate shows a non-stoichiometric (de)hydration behavior. Unexpectedly, the water molecules are not located in structural channels but at isolated-sites of the host framework, which is counterintuitively for a hydrate with non-stoichiometric behavior. The water-DDS interactions were estimated to be weaker than water-host interactions that are commonly observed in stoichiometric hydrates and the lattice energies of the isomorphic dehydration product (hydrate structure without water molecules) and (form **III**) differ only by ~1 kJ mol^−1^. The computational generation of hypothetical monohydrates confirms that the hydrate with the unusual DDS:water ratio of 3:1 is more stable than a feasible monohydrate structure. Overall, this study highlights that a deeper understanding of the formation of hydrates with non-stoichiometric behavior requires a multidisciplinary approach including suitable experimental and computational methods providing a firm basis for the development and manufacturing of high quality drug products.

## Introduction

The vast majority of drugs is formulated and administered in a solid (mostly crystalline) form, since this aggregation state assures the highest chemical and storage stability of the drug compound. However, a drug compound may occur in a variety of different solid state forms, which is subsumed under the general term “polymorphism” comprising one component forms (polymorphs, amorphous form) and multicomponent phases (hydrates, solvates, co-crystals). The statement “*Many people think that polymorphism and solid state chemistry is the hardest thing to get right in drug development*” (Byrn, 2004) clearly reflects on the challenges encountered and efforts to be undertaken in pre-formulation to guarantee that the best solid form is used in a drug formulation. The molecular structure of a drug compound determines its biological/pharmacological properties and is thus an invariant, i.e., cannot be changed in order to optimize the physicochemical and biopharmaceutical properties of a drug. The only strategy to improve such properties at the molecular level is the formation of bioreversible derivatives of the drug compound (prodrugs), which are transformed to the active molecules by metabolic principles in the organism (Rautio et al., 2008, 2017). The molecular features of a drug (molecular size, shape, flexibility, hydrogen bond donors/acceptors, etc.,) determine the potential of a drug to occur in different “supramolecular” states (solid state forms) which may exhibit significantly different physicochemical properties that are critical for the adjustment of an optimal performance of a pharmaceutical product. The most critical parameters are equilibrium solubility and dissolution rate but also differences in density, hardness, melting point, mechanical strength, chemical stability etc. may affect manufacturing processes and are relevant for shelf-life stability and finally the bioavailability of a final dosage form. Thus, identifying solid state forms of a drug and understanding their phase relationships, interconversion pathways and properties is a key concern in modern drug development (Byrn et al., 1999; Bernstein, 2002; Hilfiker, 2006; Brittain, 2009). Multiple solid forms, including salts, co-crystals and solvates, have been found for 90% of molecules (Stahly, 2007) and therefore, considerably extend the range of solid form options available for delivering drugs.

The past experience of late-appearing, more stable forms, as in the case of ritonavir (Chemburkar et al., 2000) or rotigotine (Perez-Lloret et al., 2013), has not only triggered the awareness of the issue of solid forms but also led to the implementation of polymorphism screenings, a survey of crystallization conditions designed to find and identify solid forms of a drug substance, as a routine in the pre-formulation phase. Experimental solid form screens may encompass up to thousands of crystallization experiments and need to be tailored to the properties of the investigated molecule (Newman, 2013; Cruz-Cabeza Aurora et al., 2015). The wide range of methods that have led to the discovery of novel forms (Llinàs and Goodman, 2008) highlight, however, that there is no standard recipe for comprehensive experimental solid form screening. Furthermore, the problem that there is no endpoint in experimental solid form screening, a computational method ensuring that all relevant forms have been found is in high demand. To this end, crystal structure prediction (CSP) on smaller pharmaceuticals has shown high promise in complementing experimental solid form screening, helping to rationalize and unify experimental observations on polymorphs, hydrates and solvates (Cruz-Cabeza et al., 2008; Campeta et al., 2010; Braun et al., 2011a, 2014a,b, 2016; Baias et al., 2013; Bhardwaj et al., 2013; Ismail et al., 2013; Kendrick et al., 2013; Price et al., 2014, 2016; Singh and Thakur, 2014; Braun and Griesser, 2016b; Price and Reutzel-Edens, 2016). The aim of an experimental polymorph screen is the identification of those solid state forms which are relevant for a product development, and the main expectation of a CSP study is the confirmation that those forms are among the lowest energy structures. Yet, computing the crystal energy landscapes of larger drug molecules including its hydrates and solvates is still too complex and computationally very (time) demanding. For multi-component systems host (drug molecule) and different guest molecules in different stoichiometric ratios would have to be considered separately.

Generating knowledge of how water (vapor) is associated with a specific material and how it affects the stability of a product is a crucial task in pre-formulation studies, because water inevitably appears in the manufacturing and storage process of pharmaceutical products. Knowledge about hydrate formation (water adducts) is of importance, as hydrates can be the most stable solid form at relevant production and storage conditions and it is well-known that at least one-third of organic (drug) molecules (Stahly, 2007; Braun, 2008; Cruz-Cabeza Aurora et al., 2015) form hydrates. A transformation to a hydrate may be unavoidable. In a hydrate the water molecules occupy regular positions in the crystal lattice of the parent substance. The water can either fill structural voids or be an integral part of the structure. Based on the moisture sorption/desorption behavior hydrates can be subdivided into two main classes (Gal, 1968; Griesser, 2006). “Stoichiometric” hydrates are regarded as molecular compounds. Dehydration always leads to a different structure or the amorphous state. “Non-stoichiometric” hydrates incorporate a range of water levels as a function of temperature and water vapor pressure. The latter often host water molecules in open structural voids that allow for reversible water uptake/release without significant changes in the crystal structure. The water in non-stoichiometric hydrates is often rather weakly bound and may interact with other components compromising the stability and performance of formulated products. Thus, knowledge of hydrate formation, moisture and temperature dependent stability is crucial for the development of a high quality fine chemical product.

**Figure 1:**
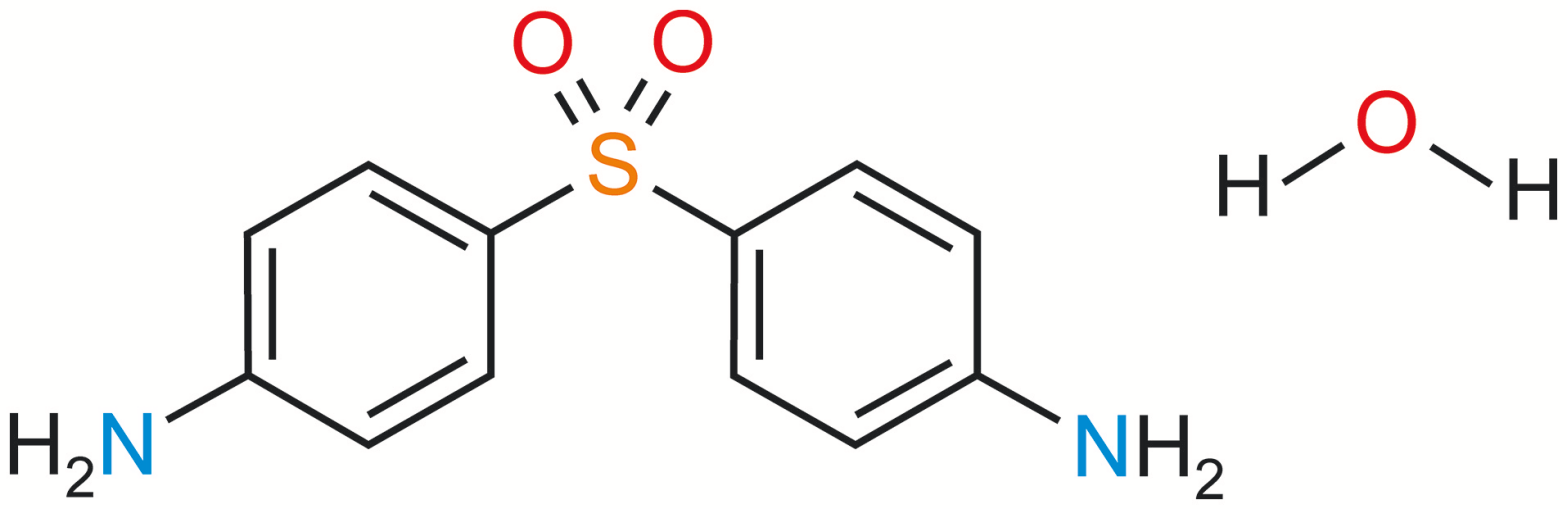
Molecular diagram of 4,4′-diaminodiphenyl sulfone (DDS, dapsone) hydrate.

Dapsone (4,4′-diaminodiphenyl sulfone; DDS, Figure 1) has been chosen in this study as a model compound for evaluating the value of computational chemistry in solid form screening and characterization of a pharmaceutical hydrate. The compound itself has been synthesized for the first time over 100 years ago (Fromm and Wittmann, 1908) and its microbial activity and therapeutic use for leprosy has already been studied in the 1940s. DDS has reinvented itself as a drug many times and has been in use for numerous indications, treatment of leprosy, dermatitis herpetiformis, malaria, prophylaxis of pneumocytosis etc. (Wolf and Orni-Wasserlauf, 2000; Wozel and Blasum, 2014). Today, it is mainly used as first-line drug in the treatment of leprosy in combination with rifampicin and clofazimine. As such, it is listed in the WHO's List of Essential Medicines (medications satisfying the priority health care needs in humans) (World Health Organization, 2017). The compound is known to be polymorphic (anhydrate forms **I**–**IV**), with form **III** being reported to be the most stable form (Brandstaetter-Kuhnert et al., 1963; Kuhnert-Brandstatter and Moser, 1979). Single crystal structures are known for anhydrate forms **III** (Dickinson et al., 1970; Deo et al., 1980; Bocelli and Cantoni, 1990; Su et al., 1992; Bertolasi et al., 1993) and **II** (Braun et al., 2017). It is also known that DDS forms solvates with dichloromethane, 1,4-dioxane and tetrahydrofuran (Babashkina et al., 2012; Lemmer et al., 2012). Furthermore, several crystal structure determinations of a hydrate with the unusual DDS:water stoichiometry of 3:1 (0.33-hydrate) have been reported (Kuz'mina et al., 1981; Bel'skii et al., 1983; Yathirajan et al., 2014). Apart from these structure reports no other information about the hydrate can be found in the literature.

The aim of this study was to unravel the molecular/structural reasons for hydrate formation in DDS, the structural and thermodynamic relationship between the **0.33-Hy** and water-free DDS forms and their interconversion pathways as a function of temperature and humidity/water activity through a combination of computational and experimental methods. A range of experimental techniques (crystallization, slurry experiments, thermal analysis and X-ray diffraction), along with CSP and pair-wise intermolecular energy calculations were applied to explore the solid forms at an atomistic level. The applied method for estimating the intermolecular interaction energies (CE-B3LYP), best described as a hybrid method, did perform surprisingly well compared to B3LYP-D2/6-31G(d,p) counterpoise-corrected energies, but in considerable less computation time (Turner et al., 2014). However, calculating water interactions in organic (drug) hydrates represents a big challenge as the balance of host (organic molecule)-host, host-water, and water-water intermolecular interactions has to be modeled accurately. Most simple water potentials have been parametrized against a wide range of liquid properties (Guillot, 2002). A potential for studying ices and amorphous water, which reparametrized the TIP4P potential to reproduce the density of several forms of ice, has been developed by Abascal et al. (2005). Very recently it has been demonstrated (in lead optimization), that accurately modeling intermolecular interactions involving water requires the incorporation of three-body terms and nanoscale treatment of the dielectric response of confined frustrated water molecules (Fernández, 2016, 2017; Fernandez and Scott, 2017). Nevertheless, in our study we decided to test the applicability of the readily available and transferable CE-B3LYP method, which was not explicitly developed for hydrate structures. We address the role of CSP in hydrate screening and modeling and investigate whether it is possible to derive information about hydrate stability and dehydration mechanism based on structural classifications and simple intermolecular interaction energy estimations (i.e., estimating the strengths of host-host, water-host and water-water interactions).

## Materials and methods

### Computational generation of the monohydrate crystal energy landscape

The global energy minimum of DDS, obtained using Gaussian09 (Frisch et al., 2009), was used in the CSP searches. 350,000 Z′ = 1 monohydrate structures were generated using CrystalPredictor2.0 (Karamertzanis and Pantelides, 2005, 2007; Habgood et al., 2015) in 48 common space groups for organic molecules (Supplementary Material). The molecules were held rigid and the lattice energy was evaluated by an exp-6 potential with atomic charges derived using the CHELPG scheme (Breneman and Wiberg, 1990) and minimized. The 10,000 lowest energy crystal structures were used as starting points for optimizing the intermolecular lattice energy (*U*_inter_), with an improved model for the intermolecular forces. This was calculated using the FIT exp-6 potential parameters (Coombes et al., 1996), the sulfur potential derived by Scheraga (Day et al., 2009) and the distributed multipoles (Stone, 2005) derived from the PBE0/6-31G(d,p) charge density using GDMA2 (Stone, 2010).

The optimal proton positions of the amino group and orientation of the phenyl groups, in all crystal structures within 15 kJ mol^−1^ of the global minimum (116 structures), were determined using the CrystalOptimizer database method (Kazantsev et al., 2011). This was done by minimizing the lattice energy (*E*_latt_), calculated as the sum of the intermolecular contributions (*U*_inter_) and the conformational energy penalty paid for distortion of the molecular geometry to improve the hydrogen bonding geometries. Conformational energy penalties (Δ*E*_intra_, with respect to the pyramidal global conformational energy minimum) and isolated molecule charge densities were computed at the PBE0/6-31G(d,p) level, for each conformation considered in the minimization of *E*_latt_. All isolated-molecule wave function calculations were performed using Gaussian09 (Frisch et al., 2009) and intermolecular lattice energies using DMACRYS (Price et al., 2010).

The 100 most stable structures (within 30 kJ mol^−1^ of the global minimum) were used as starting points for periodic electronic structure calculations. The DFT-D calculations were carried out with the CASTEP plane wave code (Clark et al., 2005) using the Perdew-Burke-Ernzerhof (PBE) generalized gradient approximation (GGA) exchange-correlation density functional (Perdew et al., 1996) and ultrasoft pseudopotentials (Vanderbilt, 1990), with the addition of a semi-empirical dispersion correction developed by Tkatchenko and Scheffler (TS) (Tkatchenko Scheffler and Scheffler, 2009). Brillouin zone integrations were performed on a symmetrized Monkhorst–Pack k-point grid with the number of k-points chosen to provide a maximum spacing of 0.07 Å^−1^ and a basis set cut-off of 780 eV. The self-consistent field convergence on total energy was set to 1 × 10^−5^ eV. Energy minimizations were performed using the Broyden–Fletcher–Goldfarb–Shanno optimization scheme within the space group constraints. The optimizations were considered complete when energies were converged to better than 2 × 10^−5^ eV per atom, atomic displacements converged to 1 × 10^−3^ Å, maximum forces to 5 × 10^−2^ eV Å^−1^, and maximum stresses were converged to 1 × 10^−1^ GPa. Isolated molecule minimizations to compute the isolated DDS and water energy (*U*_gas_) were performed by placing a single molecule in a fixed cubic 35 × 35 × 35 Å^3^ unit cell and optimized with the same settings as used for the crystal calculations.

The experimental hydrate, lower hydrates and forms **II** and **III** of DDS, as well as other selected hydrates (Supplementary Material), were minimized with CASTEP and the same settings were used as described for generating the monohydrate crystal energy landscape.

### Crystal explorer calculations

The pair-wise energy contributions to **0.33-Hy** and other well-characterized hydrate structures, have been calculated using CrystalExplorer V17 (Turner et al., 2014, 2015; Mackenzie et al., 2017). The optimized atomic positions (PBE-TS) have been used in all subsequent intermolecular interaction energy calculations. The model energies have been calculated between all unique nearest neighbor molecular pairs. The used model (termed CE-B3LYP) uses B3LYP/6-31G(d,p) molecular wave functions calculated by applying the molecular geometries extracted from the crystal structures. This approach uses electron densities of unperturbed monomers to obtain four separate energy components: electrostatic (*E*_E_), polarization (*E*_P_), dispersion (*E*_D_), and exchange-repulsion (*E*_R_). Each energy term was scaled independently to fit a large training set of B3LYP-D2/6-31G(d,p) counterpoise-corrected energies from both organic and inorganic crystals. The CE-B3LYP energies reproduced the training set energies with a mean absolute deviation of ~1 kJ mol^−1^ (Turner et al., 2014).

### Conformational analysis

Conformational energy scans were performed at the B3LYP/6-31G(d,p) level of theory using Gaussian09 (Frisch et al., 2009), allowing the two torsion angles defining the position of the phenyl rings, C–C–S–C, to rotate by 360° in 20° steps.

### Materials and preparation of DDS hydrate

Dapsone form **III** (purity 97%) was purchased from Aldrich. The obtained sample was recrystallized from a hot-saturated methanol solution. The solid product was isolated by filtration and consisted of form **III**. The organic solvents used were all of analytical grade and purchased from Aldrich or Fluka.

DDS **0.33-Hy** was prepared as follows: (i) a slurry of DDS form **III** in water was stirred in the temperature range from 10 to 30°C for 1 week. The suspension was filtered and the solid was stored at ambient conditions. (ii) A hot saturated solution of form **III** in water (close to the boiling point) was cooled to room temperature (RT). Within 2 days large elongated **0.33-Hy** crystals were obtained.

### Thermal analysis

For hot-stage thermomicroscopic (HSM) investigations a Reichert Thermovar polarization microscope, equipped with a Kofler hot-stage (Reichert, A), was used. Photographs were taken with an Olympus DP71 digital camera (Olympus, A).

Differential Scanning Calorimetry (DSC) thermograms were recorded on a Diamond DSC (Perkin-Elmer Norwalk, Ct., USA) controlled by the Pyris 7.0 software. Using a UM3 ultramicrobalance (Mettler, Greifensee, CH), samples of ~5–7 mg were weighed into perforated or sealed aluminum pans. The samples were heated using rates in between 1 and 20°C min^−1^ and cooled using a rate of 5 or 10°C min^−1^ with dry nitrogen as the purge gas (purge: 20 mL min^−1^). The instrument was calibrated for temperature with pure benzophenone (mp 48.0°C) and caffeine (236.2°C), and the energy calibration was performed with indium (mp 156.6°C, heat of fusion 28.45 J g^−1^). The errors on the stated temperatures (extrapolated onset temperatures) and enthalpy values were calculated at the 95% confidence interval (CI) and are based on at least five measurements.

Thermogravimetric Analysis (TGA) was carried out with a TGA7 system (Perkin-Elmer, Norwalk, CT, USA) using the Pyris 2.0 Software. Approximately 7–10 mg of sample was weighed into a platinum pan. Two-point calibration of the temperature was performed with ferromagnetic materials (Alumel and Ni, Curie-point standards, Perkin-Elmer). Heating rates of 5 and 10°C min^−1^ were applied and dry nitrogen was used as a purge gas (sample purge: 20 mL min^−1^, balance purge: 40 mL min^−1^).

### Powder X-ray diffraction (PXRD)

PXRD patterns were obtained using an X'Pert PRO diffractometer (PANalytical, Almelo, NL) equipped with a θ/θ coupled goniometer in transmission geometry, programmable XYZ stage with well plate holder, Cu-Kα_1, 2_ radiation source with a focusing mirror, a 0.5° divergence slit, a 0.02° Soller slit collimator on the incident beam side, a 2 mm antiscattering slit, a 0.02° Soller slit collimator on the diffracted beam side and a solid state PIXcel detector. The patterns were recorded at a tube voltage of 40 kV and tube current of 40 mA, applying a step size of 2θ = 0.013° with 200 s per step in the 2θ range between 2° and 40°. For non-ambient RH measurements, a VGI stage (VGI 2000M, Middlesex, UK) was used.

The diffraction patterns were indexed using the first 20 peaks with DICVOL04 and the space group was determined based on a statistical assessment of systematic absences (Markvardsen et al., 2001) as implemented in the DASH structure solution package (David et al., 2006). Pawley fits (Pawley, 1981) and Rietveld refinements (Rietveld, 1969) were performed with Topas Academic V5 (Coelho, 2012). The background was modeled with Chebyshev polynomials and the modified Thompson-Cox-Hastings pseudo-Voigt function was used for peak shape fitting. For the Rietveld refinements the DDS and water molecules were treated as rigid body molecules using the PBE-TS optimized conformations of the **0.33-Hy** structure.

### Gravimetric moisture sorption/desorption experiments

Moisture sorption and desorption studies were performed with the automatic multisample gravimetric moisture sorption analyser SPS23-10μ (ProUmid, Ulm, D). Approximately 500–750 mg of sample was used for each analysis. The measurement cycles were started at 60% with an initial stepwise desorption (decreasing humidity) to 0%, followed by a sorption cycle (increasing humidity) up to 90% relative humidity (RH), a desorption cycle to 0% RH and a final sorption cycle to 90% RH. The RH changes were set to 2% and the equilibrium condition for each step was set to a mass constancy of ± 0.001% over 60 min and a maximum time limit of 48 h per step.

### Water activity measurements (slurry method)

DDS form **III** was stirred (500 r.p.m.) in 1.5–2.5 mL of each methanol and water mixture [each containing a different mole fraction of water corresponding to a defined water activity Zhu et al., 1996, Supplementary Material] at 25.0 ± 0.1°C for 21 days. Samples were withdrawn, filtered and the resulting phase was determined using PXRD.

## Results and discussion

### Computational screening for DDS monohydrates

**Figure 2:**
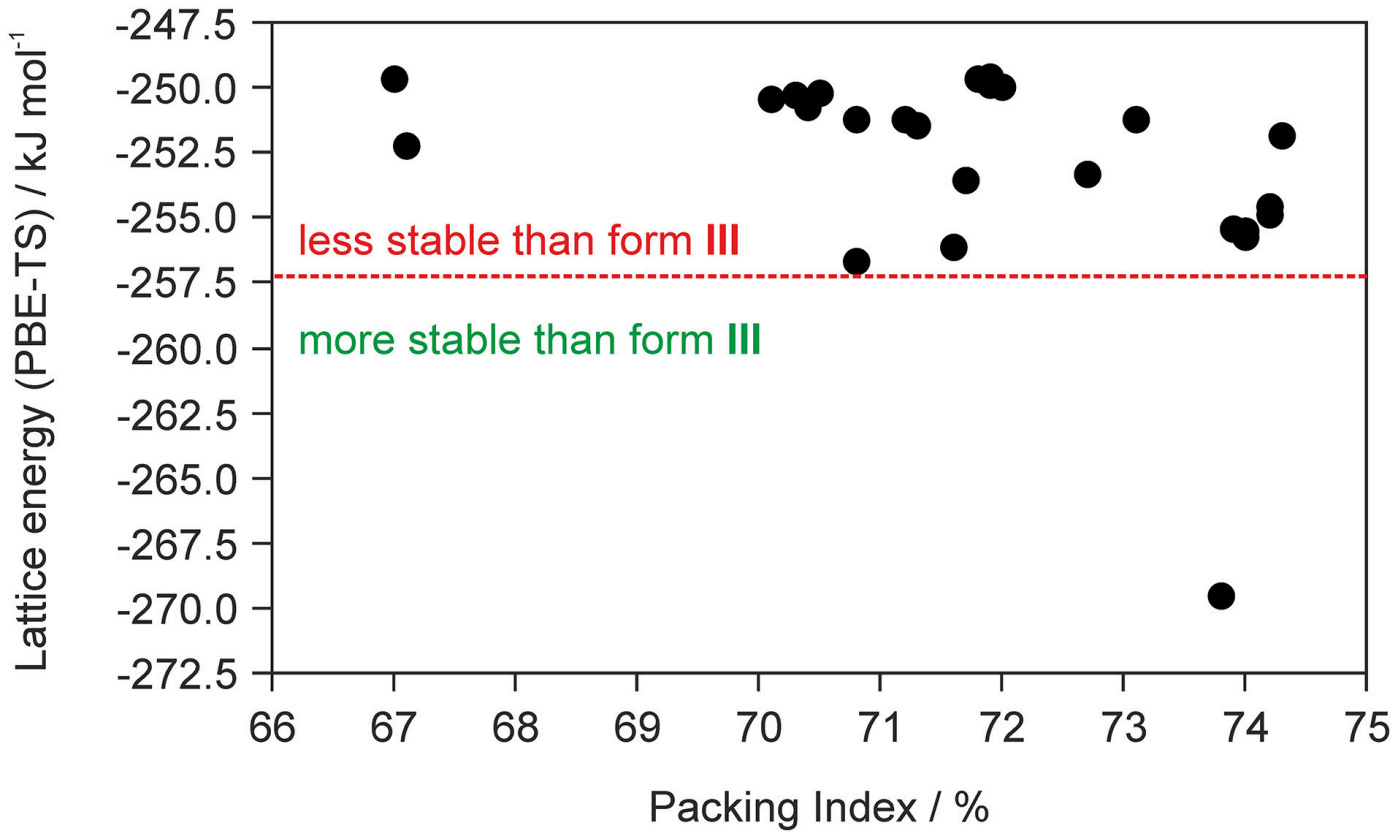
Summary of crystal structure prediction for DDS monohydrate (Z′ = 1), with each symbol denoting a crystal structure by its lattice energy and packing index. The vertical red dotted line separates the monohydrate structure that was calculated to be more stable than form III and ice from other computed hydrate structures that are less stable.

The fact that the asymmetric unit of **0.33-Hy** (Kuz'mina et al., 1981; Bel'skii et al., 1983; Yathirajan et al., 2014) consists of four crystallographically distinct molecules (three DDS and one water) makes CSP studies for the experimental stoichiometry too time-consuming. Therefore, we decided to generate the monohydrate crystal energy landscape (one DDS and one water molecule) with the aim to estimate whether water molecules can compete against the DDS-DDS intermolecular interactions and form strong DDS-water contacts. In Figure 2 the computed monohydrate structures are plotted according to lattice energy, which equals the energy that would be required to separate the molecules to infinity, against packing index.

To estimate whether any of the hypothetical monohydrate structure is competitive in energy with form **III**, we compared the lattice energy of the hydrate (*E*_latt_-Hy) to the lattice energies of the anhydrate (*E*_latt_-III) and ice (E_latt_-ICE). If *E*_latt_-Hy < *E*_latt_-III + E_latt_-ICE (we assume that hydrate formation is thermodynamically driven), then the hydrate is more stable than the anhydrate. Using the lattice energies of **0.33-Hy**, form **III** (Table 1) and a value of −59 kJ mol^−1^ (Whalley, 1957, 1976) for ice, as the used functional is known to overbind the ice crystal structures (Thierfelder et al., 2006; Beran and Nanda, 2010), then only one structure, 01_1963, was calculated to be more stable than **III**. The most stable hypothetical monohydrate was estimated to be 12.38 kJ mol^−1^ more stable than form **III**, which is a respectable potential energy differences (Δ_trs_*U*) for a monohydrate with respect to an anhydrate. Thus, the CSP study clearly indicates hydrate formation.

**Table 1:** Lattice energy calculations (*E*_latt_) of **0.33-Hy**, 01_1963, forms **II** and **III** and the isomorphic dehydrate structure (**0.33-Hy** without water molecules, **Hy**_dehy_) and potential energy differences (Δ_trs_*U*) with respect to form **III**.

**Form**	***E***_latt_/**kJ mol^−1^**	Δ_trs_***U***_x−III_/**kJ mol^−1^**
**0.33-Hy**	−222.95	15.37^a^
**Hy**_dehy_	−197.26	−0.90^b^
01_1963	−269.54	12.38^a^
Form **II**	−194.92	−3.25^b^
Form **III**	−198.16	0^b^

a *Calculated according to: –Δ_trs_U_x−**III**_ = E_latt_-x – (E_latt_-**III** + E_latt_-ICE)*.

b *–Δ_trs_U_x−**III**_ = E_latt_-x– E_latt_-**III***.

Furthermore, Δ_trs_*U* for the **0.33-Hy** to form **III** was calculated to be 15.37 kJ mol^−1^, indicating that the experimental hydrate is 3 kJ mol^−1^ more stable than the computed lowest energy monohydrate structure and rationalizing why the stable 0.33-hydrate and not a monohydrate is formed experimentally.

### Experimental screening for DDS hydrate(s)

To confirm that **0.33-Hy** is indeed the stable DDS hydrate form and that 01_1963 is not a yet undiscovered monohydrate we subjected DDS to an experimental hydrate screening program. Evaporative crystallization experiments of DDS from a saturated (20°C) aqueous solution, as well as cooling crystallization experiments from hot (boiling) saturated solutions in water at 5°, 25°, 50°, and 75°C resulted in **0.33-Hy** crystals in the form of elongated plates. In contrast, evaporation experiments of a hot-saturated solution of DDS in water resulted in a mixture of **0.33-Hy** and form **III**. Slurry experiments in water, isothermal or cycling between 5° and 50°C, always yielded the **0.33-Hy**.

Another successful way to produce hydrates are moisture sorption experiments. Therefore, form **III** and **V** (see section Moisture Dependent Stability of the Hydrate) were subjected to automated and manual water vapor sorption experiments. Neither form **III**, nor form **V** showed a transformation in the RH range up to 90%. Furthermore, no transformation was observed in long-time storage experiments of the two anhydrous DDS forms over saturated KOAc (24% RH), K_2_CO_3_ (43% RH), NaCl (75% RH), KNO_3_ solutions (92% RH) or water (100% RH) within 3 months (end of experiments) at RT and 8°C. Similarly, also **0.33-Hy** did not dehydrate or transform to another hydrate if stored under the same conditions over the same time period.

### Characterization of the DDS hydrate

#### DDS hydrate structure and intermolecular interaction energies

To identify the key interactions in the DDS hydrate the pair-wise CE-B3LYP intermolecular energies were estimated starting from the PBE-TS optimized hydrate with the CSD Refcode ANSFON02 (Yathirajan et al., 2014). The intermolecular energies are subdivided into classical electrostatic (*E*_E_), polarization (*E*_P_), dispersion (*E*_D_) and exchange-repulsion energies (*E*_R_) and can be graphically represented by their “energy frameworks” (Turner et al., 2014, 2015; Mackenzie et al., 2017).

**Figure 3:**
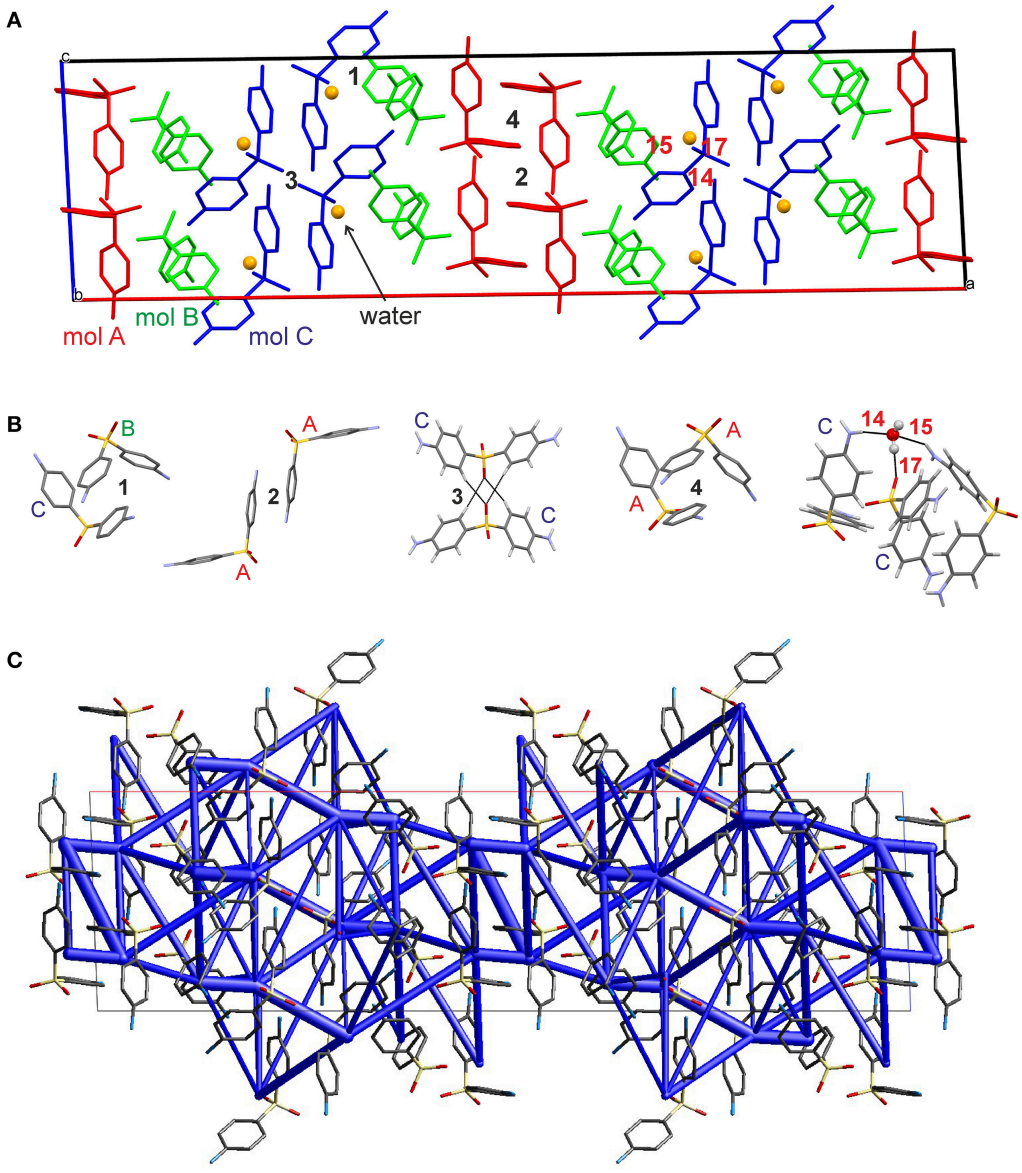
**(A)** Packing diagram of DDS **0.33-Hy** (ANSFON02, Yathirajan et al., 2014) viewed along the crystallographic *b* axis. The symmetry independent molecules are color coded and hydrogen atoms have been omitted for clarity. Numbers denote key pair-wise intermolecular interactions, which are enlarged in **(B)** and listed in Table 2. Hydrogen bonding is indicated with black lines. **(C)** Energy frameworks (total energy) for **0.33-Hy**, viewed along the crystallographic *b* axis. The energy scale factor is 80, and interaction energies with magnitudes smaller than 15 kJ mol^−1^ have been omitted. For additional views see Supplementary Material.

The hydrate crystallizes in the monoclinic space group *C*2/*c* with three DDS and one water molecule in the asymmetric unit, rationalizing the 3:1 stoichiometry (Kuz'mina et al., 1981; Bel'skii et al., 1983; Yathirajan et al., 2014). The three crystallographically independent DDS molecules (color coded in the packing diagram shown in Figure 3A) exhibit very similar conformations. The first DDS molecule (mol A, shown in red in Figure 3A) does not show any interaction with the hydrate water (Table 1), but forms two strong intermolecular interactions with itself, denoted with 2 and 4, mediated by inversion and 2-fold symmetry, respectively (Figure 3B). Furthermore, mol A forms classical hydrogen bonded interactions with neighboring DDS molecules (interactions 5, 7, and 11; Table 2). In contrast to interactions 2 and 4, which have dispersion as the strongest contributor to the energy (π···π stacks), Coulomb interactions are the reason for the stability of the latter three (also true for 3, 6, 8, 9, 14, 15, 16, and 21). Molecule B (green) and C (blue) form the strongest pair-wise interaction (1, Figure 3B), which can be related to interaction 4. The third most stable interaction (3) involves mol C and is formed of four C–H···O close contacts. The strongest classical hydrogen bonded interaction (5, Table 2), N–H···O, is significantly less stable than interactions 1–4.

**Table 2:** Pair-wise intermolecular interaction energies^a^ (Figure 3C) of DDS **0.33-Hy**.

**ID^b^**	**Mol^c^**	**Mol^c^**	**N^d^**	**Symmetry operation**	**d^e^**	***E*_E_**	***E*_P_**	***E*_D_**	***E*_R_**	***E*_tot_**	**Interaction**
					**Å**	**kJ mol**^**−1**^					
1	mol B	mol B	1	–	3.84	−16.0	−5.2	−76.1	53.7	−53.9	π···π
2	mol A	mol A	1	–*x*, –*y*, –*z*	8.37	−35.4	−6.6	−39.7	38.0	−53.3	
3	mol C	mol C	1	–*x*+1/2, –*y*+1/2, –*z*	5.72	−47.1	−15.7	−29.0	54.0	−53.3	4xC–H···O
4	mol A	mol A	1	–*x, y*, –*z*+1/2	3.58	−11.5	−5.4	−89.5	75.9	−47.2	π···π
5	mol A	mol C	1	–	8.83	−33.6	−9.6	−12.7	32.4	−33.7	N–H···O
6	mol C	mol C	2	–*x*+1/2, *y*+1/2, –*z*+1/2	8.31	−28.1	−7.9	−13.6	22.4	−33.5	N–H···O
7	mol A	mol A	1	–	9.03	−29.5	−8.4	−10.1	23.5	−31.7	N–H···O
8	mol B	mol B	2	*x*, –*y*, z+1/2	8.46	−28.7	−7.2	−10.4	21.5	−31.5	N–H···O
9	mol B	mol B	2	*x*, –*y, z*+1/2	8.84	−27.1	−10.1	−13.7	29.1	−30.1	N–H···O
10	mol B	mol A	1	–	6.58	−16.6	−6.4	−32.0	32.9	−29.9	
11	mol A	mol A	2	*x*, –*y, z*+1/2	9.53	−33.1	−10.8	−14.9	42.9	−29.6	N–H···O
12	mol A	mol A	2	*x*, –*y, z*+1/2	7.86	−15.4	−4.7	−19.4	20.6	−23.9	N–H···N
13	mol B	mol C	1	–	9.2	−12.2	−4.5	−16.3	10.7	−23.8	
14	mol C	water	1	–	7.08	−25.9	−6.2	−6.1	23.7	−22.5	N–H···O_W_
15	mol B	water	1	–	6.62	−16.7	−2.9	−5.7	8.6	−19.5	N–H···O_W_
16	mol C	mol C	1	–*x*+1/2, –*y*+1/2, –*z*	11.23	−9.4	−2.2	−11.0	2.8	−19.4	
17	mol C	water	1	–	5.25	−33.8	−7.2	−4.4	41.4	−19.2	O_W_-H···O
18	mol B	mol C	1	–	10.37	−20.0	−5.1	−12.6	27.5	−18.9	N–H···N
19	mol C	mol C	2	*x*, –*y, z*+1/2	8.13	−6.5	−3.8	−9.3	3.9	−15.4	

a Electrostatic (E_E_), polarization (E_P_), dispersion (E_D_) and exchange-repulsion energy (E_R_) contributions. E_tot_ = k_E_ E_E_ + k_P_ E_P_ + k_D_ E_D_ + k_R_ E_R_, with k being scale factors (Mackenzie et al., 2017)

b Interaction ID;

c molecule according to Figure 3A;

d *N – number of times interaction is present*.

e *Centroid distances*.

The water molecule, interacting only with mol B and mol C, forms three hydrogen bonds, two N–H···O_water_ and one O_water_ –H···O. Thus, the water molecule shows the water environment type DAA (Infantes et al., 2007), with A corresponding to hydrogen bonding acceptor and D to hydrogen bonding donor. According to Morris and Rodriguez-Hornedo (Morris and Rodriguez-Hornedo, 1993; Brittain et al., 2009) the DDS hydrate can be classified as an isolated-site hydrate, which retains water in segregated pockets in the crystal structure. The strongest pair-wise water-DDS interaction was calculated to be −22.5 kJ mol^−1^, which is distinctly weaker than the strongest pair-wise DDS-DDS interaction (−53.9 kJ mol^−1^).

**Figure 4:**
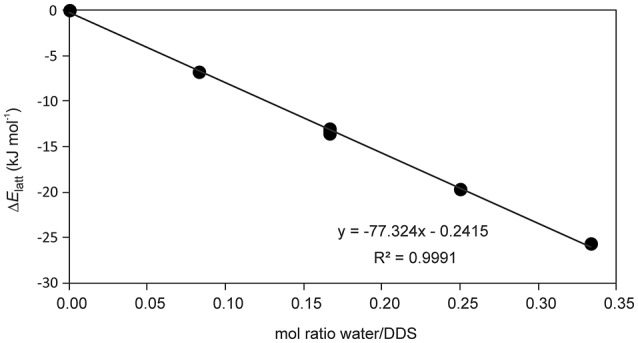
Energetic contribution (Δ*E*_latt_, see Supplementary Table 5) of the water to the DDS hydrate structure in dependency of water occupancy (mol ratio water/DDS) in **0.33-Hy**.

The presence of an isolated-site hydrate could be confirmed by calculating the total energy of the hydrate and lower hydrate structures thereof, i.e., structures which were generated by systematically removing water molecules from the packing presented in Figure 3 (using the P1 cell). Figure 4 shows that a plot of the energy contributions from the water molecules to the hydrate structure vs. the water content gives a linear relationship. This clearly indicates that the water molecule interacts solely with DDS molecules in the **0.33-Hy**, which is a characteristic feature of an isolated-site hydrate.

#### Temperature dependent stability of the hydrate

**Figure 5:**
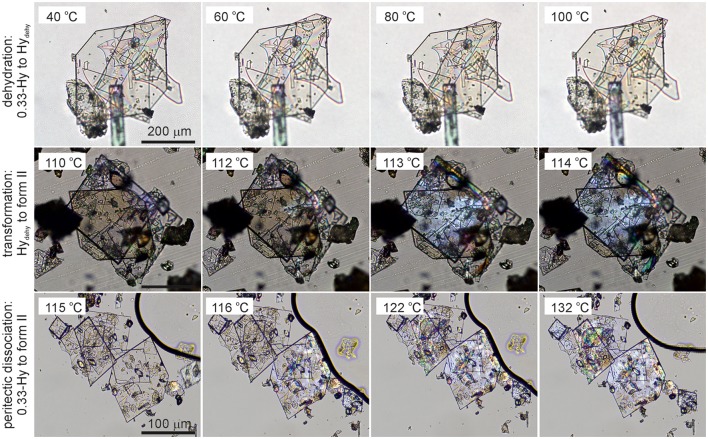
Photomicrographs of DDS **0.33-Hy**. Dehydration in the temperature range 40°-100°C, **Hy**_dehy_ to form **II** transformation in the temperature range 110°-114°C, and peritectic dissociation (crystals embedded in high-viscosity silicon oil) of **0.33-Hy** to form **II** in the temperature range 115°−132°C.

**Figure 6:**
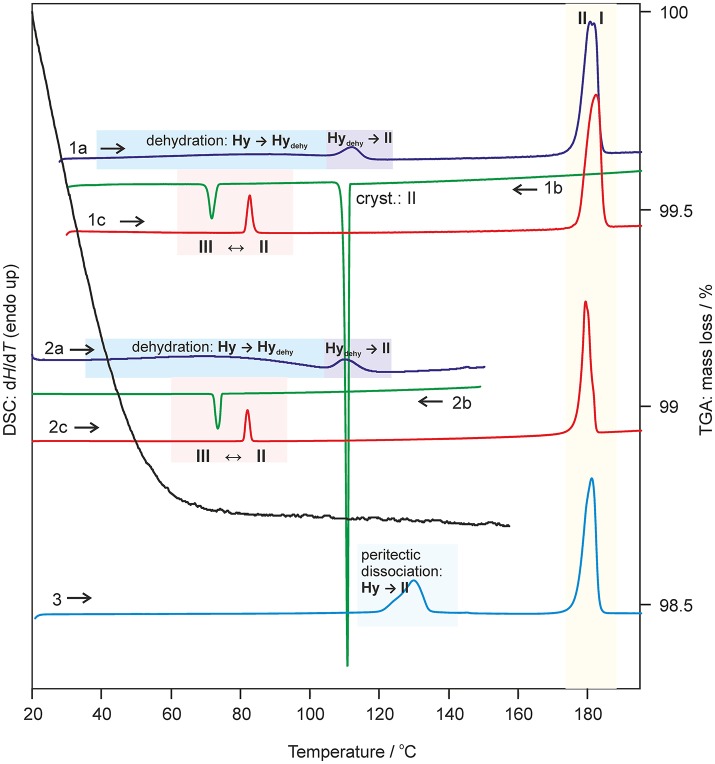
Selected DSC and TGA thermograms of DDS **0.33-Hy**, recorded using a heating/cooling rate of 10°C min^−1^. DSC curves 1 and 2 were measured using perforated DSC crucibles and curve 3 using a hermetically sealed 3bar DSC crucible. I, II, III-anhydrous forms **I**, **II**, and **III**; Hy, **0.33-Hy**; dehy, **Hy**_dehy_.

Key information for handling and storing hydrates is knowledge about temperature- and moisture-dependent stability. The dehydration process of **0.33-Hy** was monitored with HSM (Figure 5), DSC and TGA (Figure 6). To investigate the impact of the atmospheric conditions on the dehydration behavior and associated processes, different experimental conditions were applied: dry and silicon oil preparations (HSM), heating of the sample in perforated or sealed DSC crucibles and using different heating rates. The obtained thermodynamic data are summarized in Table 3.

**Table 3:** Thermochemical data of DDS solid forms (T, temperature; Δ*H*, enthalpy) obtained starting from the hydrate (**Hy: 0.33** mol water/mol DDS) compared with the CE-B3LYP energy estimation.

**Process**	**DSC**		**CE-B3LYP**
	**T/°C**	**Δ*H*/kJ mol^−1^**	**kJ mol^−1^**
**DEHYDRATION**			
Hy^a^ → Hy_dehy_	40–90	10.60 ± 0.10	
Hy → Hy_dehy_		16.95 ± 0.13^b^	
Contribution of water to Hy structure			13.85
**TRANSFORMATION**			
Hy → Hy_dehy_	40–90	2.95 ± 0.09	2.98^c^
Hy_dehy_ → II	103.6 ± 2.8	1.08 ± 0.05	
II → III	75.2 ± <0.1	−2.02 ± 0.08	
III → II	81.5 ± 0.2	2.06 ± 0.07	
**PERITECTIC DISSOCIATION**			
Hy → II	126.5 ± 0.5	4.98 ± 0.53	

a *Referred to a hydrate with 0.18 mol water/mol DDS*.

b *Estimated form 0.18-Hy*.

c *13.85 kJ mol^-1^–10.87 kJ mol^-1^*.

The dehydration of **0.33-Hy** occurs in the temperature range from 40 to 90°C. With HSM (dry preparation, Figure 5) hardly any change is observed during the dehydration process of the **0.33-Hy** to the isostructural dehydrate (**Hy**_dehy_). However, with TGA and DSC the dehydration is well observable. Under N_2_ purge (TGA) the dehydration process starts immediately and a mass loss of ~0.3 mol of water per mol DDS was determined. In DSC investigations (1a, 2a), the dehydration appears as a broad endothermic event which partly overlaps with a second endothermic process at a heating rate of 10°C min^−1^. Using lower heating rates (not shown) the two thermal events can be separated and the heat of dehydration, Δ_dehy_*H*_Hy−dehy_ of 10.60 kJ mol^−1^(sample contained 0.18 mol water per mol DDS) was determined. The known enthalpy value for the vaporization of water at the dehydration temperature (*T*_dehy_ ≈ 60°C, Δ_vap_*H* H_2_O = 42.482 kJ mol^−1^ (Riddick and Bunger, 1986) can be subtracted from the measured heat of dehydration (Δ_dehy_*H*), according to Equation (1), resulting in an estimation of the heat change (Δ_trs_*H*) upon hydrate to anhydrate transformation. The enthalpy of this reaction was calculated to be 2.95 ± 0.09 kJ mol^−1^ (Table 3).

(1) $$\Delta_{trs}H_{Hy-dehy}=\Delta_{dehy}H_{Hy-dehy}-0.18\cdot \Delta_{vap}H_{H2O}$$

The second endotherm of the DSC traces (perforated crucibles) with an onset temperature of 103.6°C corresponds to the solid-solid phase transformation of **Hy**_dehy_ to form **II** (1.08 kJ mol^−1^). In HSM investigations an increase in birefringence is observable during the transformation process. Upon further heating, form **II** melts at 177.2°C (1a) and concomitantly form **I** crystalizes, which then melts at 179°C. Upon cooling the melt of DDS (1b) spontaneous crystallization of form **II** occurs around 110°C. The presence of form **II** is confirmed by the occurrence of the exothermic event at 75°C (cooling curve), indicating the transformation of form **II** to form **III**. The measured enthalpy value of −2.02 kJ mol^−1^ agrees with the enthalpy value of the transformation **III** → **II** (2.06 kJ mol^−1^), which can be determined on reheating. A more detailed study of this transformation has been reported just recently by us (Braun et al., 2017). In a separate experiment, the DSC heating run of **0.33-Hy** was stopped above the **Hy**_dehy_ → **II** transition peak (2a) and the subsequent cooling curve shows the exothermic **II** → **III** transition (2b). The temperature range and enthalpy of this spontaneous transition confirm unambiguously that mainly form **II** is present after the hydrate is heated to about 150°C. Form **III** transforms back to form **II** at 81°C (1c and 2c) just about 6°C above the **II** → **III** transition peak highlighting the weak kinetic control of this reversible solid-solid transformation.

By embedding the hydrate crystals into high viscosity silicon oil (HSM, Figure 5) or using hermetically sealed DSC crucibles (3, Figure 6) the peritectic dissociation process of **0.33-Hy** to form **II** can be observed or recorded around 125°C, respectively. A fast nucleation and growth process of form **II** occurs, thus no clear melting process is observable by HSM and the phase transition is mainly indicated by an increase in birefringence (Figure 5). The measured heat of ~5 kJ mol^−1^ can be related to the **0.33-Hy** to form **II** transformation, but also includes an unknown contribution from the enthalpy of solution of a fraction of the dehydration product in the liberated water. Due to the low water solubility of DDS and the low water stoichiometry of the hydrate the measured **0.33-Hy** to form **II** enthalpy is only slightly higher than the sum of the heats of **0.33-Hy** to **Hy**_dehy_ and **Hy**_dehy_ to form **II** transformations of ~4 kJ mol^−1^. Thus for DDS, it is possible to estimate the **0.33-Hy** to form **II** transition enthalpy directly in a hermetically sealed DSC crucible.

#### Moisture dependent stability of the hydrate

**Figure 7:**
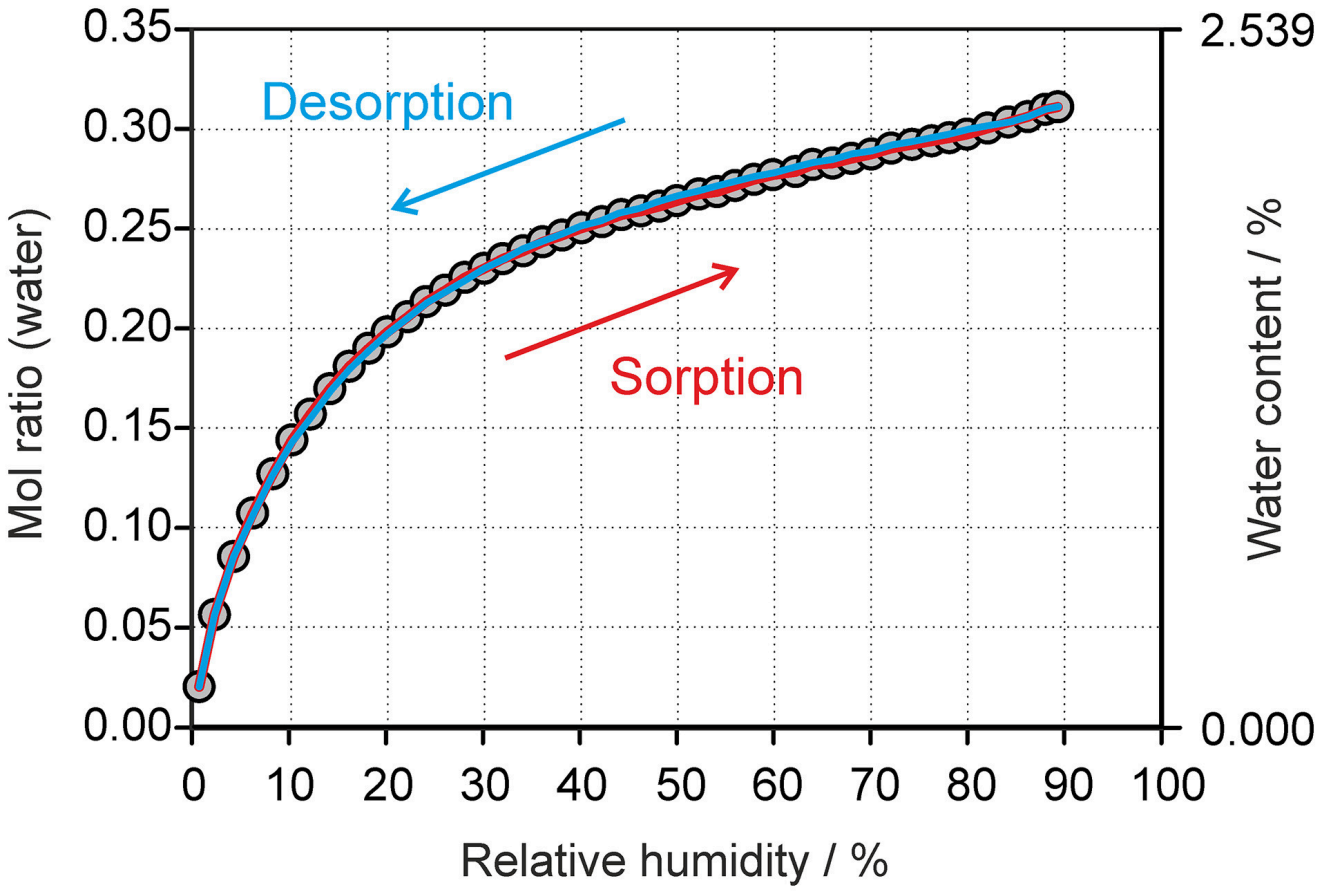
Gravimetric moisture sorption/desorption isotherms of DDS **0.33-Hy** at 25°C. Circles represent data points recorded at equilibrium conditions (see experimental section).

The moisture sorption/desorption experiments of **0.33-Hy** (Figure 7) clearly indicate a non-stoichiometric hydration/dehydration behavior. The isotherm shows a continuous course and the water content of the hydrate adjusts quickly to a specific value if the RH is altered. It is particularly striking that the sorption and desorption isotherms are superimposable, i.e., that there is no hysteresis between the sorption and desorption curve. This fact and the short time to reach the equilibrium water content on changing RH suggests that the diffusion of water molecules into or out of the structure occurs without special constraints and without significant changes of the DDS framework. This observation is even more surprising because the water molecules are located at isolated-sites in the **0.33-Hy** structure (Figure 3A) and not in open structure voids (channels, layers), which is the commonly expected feature for hydrates with a non-stoichiometric dehydration behavior.

The automated gravimetric moisture sorption/desorption analysis of **0.33-Hy** was complemented with longer-term drying experiments at 0% RH (storage over P_2_O_5_) at 25°, 50°, and 75°C to investigate whether **Hy**_dehy_ transforms to another DDS anhydrate polymorph. **Hy**_dehy_ is stable for at least 3 weeks at 25°C and for at least 10 days at 50°C. At 75°C the transformation to form **III** starts within 1 week at 0%. No new polymorph emerged in the drying studies.

**Figure 8:**
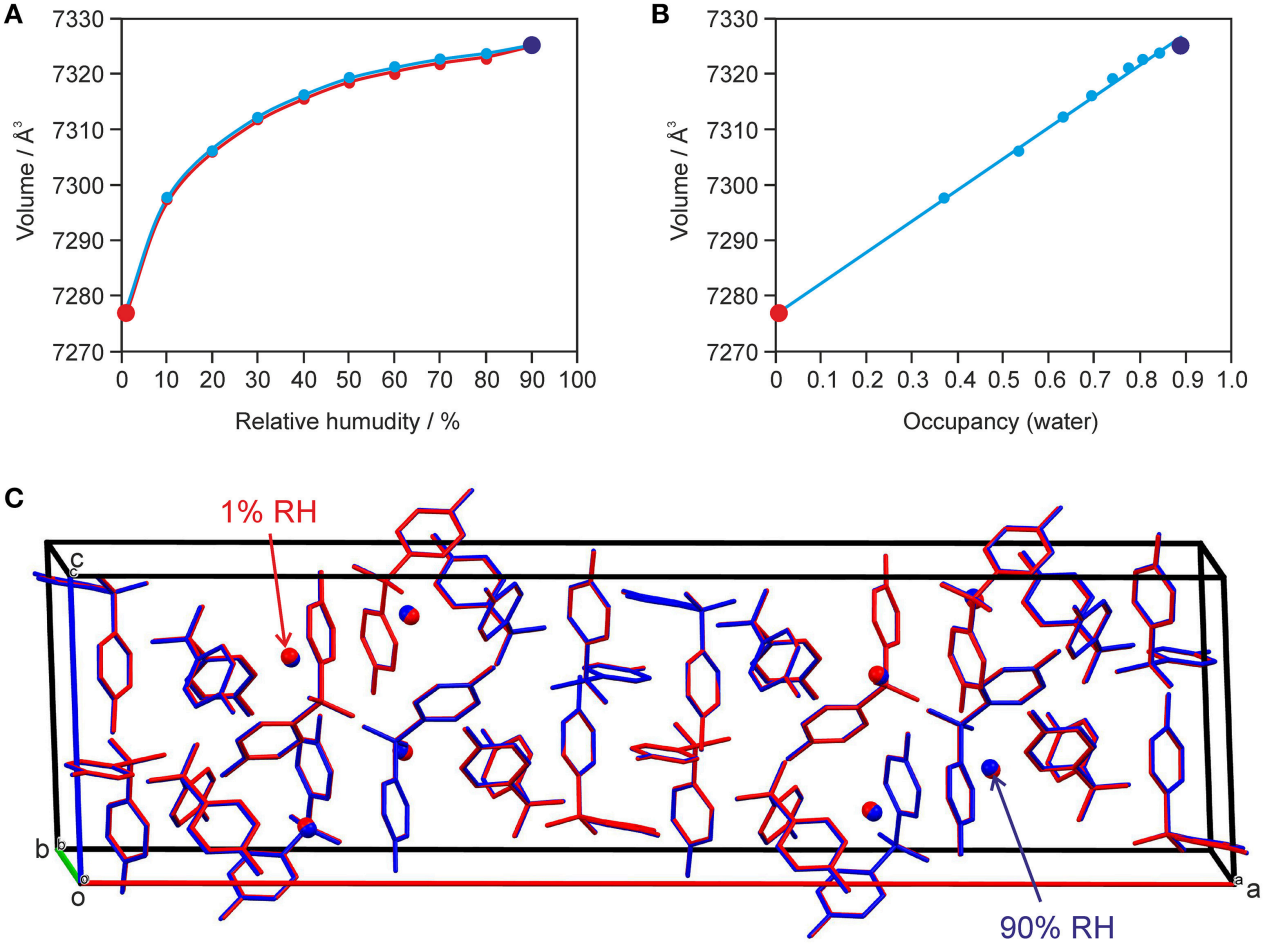
Data derived from moisture-dependent PXRD measurements and Rietveld refinement showing **(A)** the change in cell volume with RH und **(B)** factional occupancy factor of the water molecule vs. cell volume in **0.33-Hy**. In **(C)** an overlay of the hydrate at 90% (in blue) and 1% RH (red) is shown. H-atoms are omitted for clarity.

The changes seen in the gravimetric moisture sorption/desorption studies (Figure 7) were correlated with structural changes to **0.33-Hy** using variable-humidity PXRD at 25°C (Figure 8, Supplementary Material for the PXRD patterns). In the case of the DDS hydrate only slight changes in peak positions and peak intensities can be observed with varying RH. Changes in lattice parameters were quantified by indexation and Rietveld refinement of the **0.33-Hy** PXRD patterns recorded at different RH values. The lattice parameters changed by max. 0.2% in the range 90% to 1%, and the cell volume by only 0.66%. Such small changes are in the range one would expect from a non-stoichiometric hydrate and they are for example of similar magnitude as measured for the non-stoichiometric hydrate HyA of brucine (Braun and Griesser, 2016a). Plotting the **0.33-Hy** cell volume in dependence of the RH (Figure 8A) perfectly reproduces the course of the sorption/desorption isotherms in Figure 7.

Using the optimized (PBE-TS) experimental structure as starting model, Rietveld refinements were performed with PXRD patterns of samples recorded in 10% RH steps during a desorption and sorption cycle. The aim of this study was to unravel whether the water position in the **0.33-Hy** varies depending on the RH conditions. Figure 8C exemplarily shows an overlay of the hydrate structures at 90 and 1% RH. The DDS molecules are superimposable and also the water shows hardly any positional variation with RH. The structures solved at different RH values differ solely in the fractional occupancy factor to which the water molecule refined to (Figure 8B). It is surprising that this method works so well even though the water is only a very minor contributor to the overall electron density of the hydrate structure. The lowest water content observed for **0.33-Hy** in the RH dependent PXRD experiments was 0.005(12) mol of water per three moles of DDS, which is in reasonable agreement with the value determined in the automatic gravimetric sorption/desorption measurements (0.02 mol water per mol DDS) determined at the same RH. No phase change was observed in the moisture dependent PXRD experiments.

**Figure 9:**
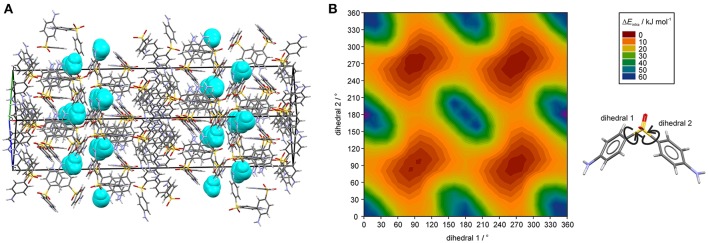
**(A)** DDS **0.33-Hy** structure (ANSFON02, Yathirajan et al., 2014), with water molecules depicted as space-fill in turquois, showing a possible water egress/ingress route parallel to [011]. **(B)** Two dimensional potential energy surface scan for DDS with respect to dihedrals 1 and 2 at the B3LYP level of theory with the 6-31G(d,p) basis set using the optimized DDS conformation.

The question remains why the water egress/ingress in the **0.33-Hy** is fast, which is not expected from a hydrate where the water molecules are located at isolated-sites. Figure 9A illustrates a possible escape route of water molecules parallel to [011]. However, this route requires cooperative movement of the diaminophenyl moieties of the DDS molecules to temporarily open up diffusion pathways, similar to that seen in hydrates of β-cyclodextrin (β-CD) (Steiner and Koellner, 1994), ciprofloxacin (Mafra et al., 2012) or DB7 (Braun et al., 2015). The potential energy surface scans of DDS reveal (Figure 9B) that considerable movement of the diaminophenyl moieties is possible with low energy cost (Δ*E*_intra_), which we assume enables the local formation of the required diffusion pathways.

**Figure 10:**
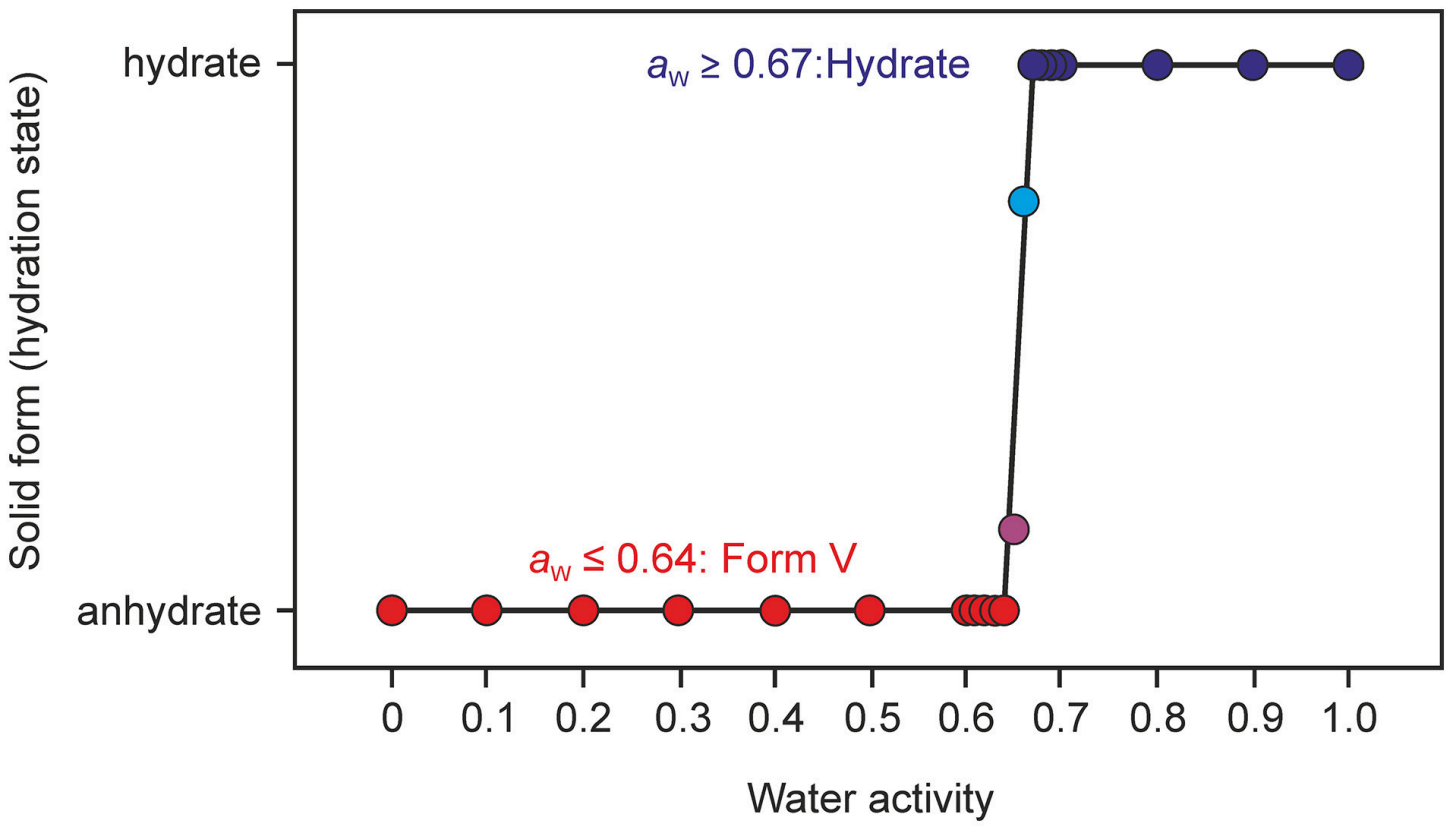
Phase diagram after equilibration for 21 days showing the stability ranges of solid phases of DDS (**form V** and the **0.33-Hy**) as a function of water activity in methanol/water mixtures at 25°C. Anhydrous form **III** was used as a starting phase; the phase identity and composition was determined with PXRD.

Sorption/desorption studies based on exposure of the solid material to various moisture conditions are controlled by kinetic parameters, which must be minimized in order to assess the thermodynamic equilibrium between the hydrate and a dehydrated state. This can be achieved for example by slurring the substance in solvents with different water activities, as has been demonstrated in previous studies (Ahlqvist and Taylor, 2002a,b; Braun et al., 2013; Braun and Griesser, 2016a). The most obvious indicator for the kinetic barrier is the hysteresis between the sorption and desorption curve observed in moisture sorption/desorption isotherms. The hysteresis can be extreme in stoichiometric hydrates and is usually small in hydrates with non-stoichiometric behavior. The isotherm of DDS **0.33-Hy** shows no hysteresis indicating that there is practically no kinetic barrier between the ingress or release processes of the water molecules to/from the crystal structure but also that the phase is maintained and no transformation to another form with different structural features occurs. To test the phase behavior under different “moisture conditions” (water activities) in solvent systems, we subjected DDS to a slurry study in methanol/water mixtures of various compositions, covering the water activity (*a*_w_) range from 0 to 1.0 (corresponding to 0 to 100% RH) in 0.01 steps and the range 0.6 to 0.7 in 0.001 steps using form **III** as the starting form (Figure 10). Surprisingly, we obtained a new anhydrous form, named form **V** hereafter, which emerged as the only stable solid phase below a water activity of 0.64. At an *a*_w_ > 0.66, **0.33-Hy** was obtained, suggesting that this hydrate is the stable form at high water activities and that the equilibrium between the DDS form **V** and the **0.33-Hy**, lies at an *a*_w_ value of ~0.655 at 25°C.

Thermal analysis and PXRD characterization confirmed that the new form **V** does not correspond to any of the four known polymorphs (Brandstaetter-Kuhnert et al., 1963; Kuhnert-Brandstatter and Moser, 1979). A thorough characterization of the new form, which is obviously the thermodynamically most stable anhydrate form at RT, and phase interrelations to the known polymorphs will be addressed elsewhere.

### Estimation of host-host, host-water, and water-water interaction energies in organic hydrates

To understand the nature and stability of a hydrate it is important to consider the location and interaction energies of the water molecules in the framework of the host structure. If water molecules are located in open structural voids (tunnels or connected pockets) the term channel hydrate (Brittain et al., 2009) is commonly used. In such hydrates the water molecules may be mobile and may readily escape through these tunnels on modest increase in temperature or decrease in relative humidity (RH). In contrast, if the water molecules are located at isolated-sites (Brittain et al., 2009), it is assumed that water egress is not as facile and requires a considerable rearrangement of the hydrate packing to allow the release of water molecules. This rearrangement results mostly in the formation of a different packing arrangement or in a partial or total collapse of the structure yielding a disordered or amorphous state upon dehydration. The non-stoichiometric behavior of hydrates, where the water content in the structure depends on the water vapor pressure of the surrounding medium (atmosphere), is normally observed in channel hydrates and not in isolated-site hydrates. However, as demonstrated above, DDS **0.33-Hy** shows clearly the typical features of an isolated-site hydrate (Figure 3A) but on the other hand shows a non-stoichiometric (de)hydration behavior (see Figure 7) which is a contradiction and questions the common relation between structural features and the stability of hydrates.

**Figure 11:**
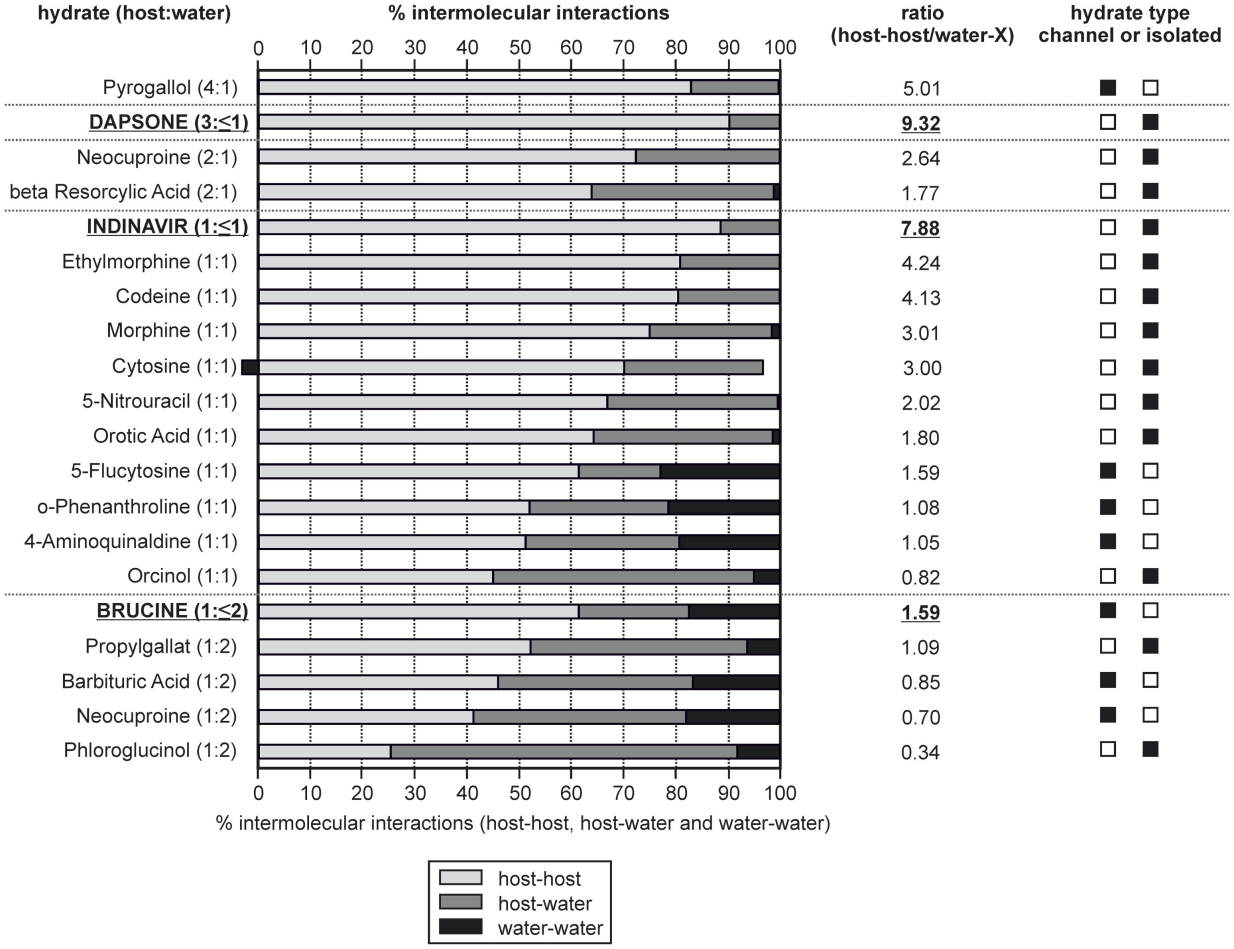
Overall contribution of intermolecular interactions in hydrate structures grouped into host-host, host-water and water-water interactions. The hydrates are ranked according to their compound:water ratio and non-stoichiometric hydrates are indicated in bold and are underlined. The ratio given was calculated as: [(host-host interactions)/(host-water and water-water interactions)].

To further clarify why the hydrate water can escape easily from the “isolated sites” in the **0.33-Hy**, without disrupting the structure, we estimated the pair-wise interaction energies for DDS and water molecules in the **0.33-Hy** structure (Table 2). The use of the CE-B3LYP energies and not a specific water potential, not considering specific effects (nanoscale dielectric responses of water) and three-body energy terms were justified by the fact that the contribution of the water to **0.33-Hy** was found to be in reasonable agreement with the experiment (Table 3). The water interactions contribute −13.85 kJ mol^−1^ to the **0.33-Hy** lattice. The CE-B3LYP energies of **0.33-Hy** (−142.87 kJ mol^−1^) and **Hy**_dehy_ (−132.00 kJ mol^−1^, optimized RT structures) differ by 10.87 kJ mol^−1^, ignoring the conformational changes which are expected to account for < 0.2 kJ mol^−1^ in the case of DDS. Thus, the sum of the interaction energies (*E*_cluster_) roughly corresponds to the lattice energy. Furthermore, we calculated the intermolecular energies for water-host, water-water and host-host molecules for a series of well characterized organic hydrate systems (pharmaceuticals and model compounds) and contrasted the values to the DDS **0.33-Hy** (Figure 11, Supplementary Material). The chosen test set consists of stoichiometric and non-stoichiometric hydrates, as well as of channel and isolated-site hydrates. This analysis should indicate, whether it is possible to assess hydrate stability and/or the dehydration mechanism from general features of the hydrate structure and not the location of the water molecules in the structure alone.

In Figure 11 the calculated interaction energies are grouped into contributions arising from host-host interactions and the incteractions including water molecules (host-water and water-water). Furthermore, the ratio between the host-host and water interactions has been calculated. The hydrates are ranked according to the compound:water ratio. For three of the chosen hydrates (dapsone, indinavir, and brucine) it is possible to remove the water molecules under maintaining the crystal structure, which is characteristic for a non-stoichiometric dehydration behavior. A grouping into channel and isolated-site hydrates is not always straight forward, in particular if more than one water molecule is present (di-, tri-hydrate, etc.,). However, based on the energetic contributions, host-water vs. water-water, such a classification is facilitated as it can be expected that in isolated-site hydrates the host-water and in channel hydrates the water-water interactions predominate, respectively. Furthermore, the sum of host-host interactions are stronger in isolated-site hydrates than in channel hydrates.

A requirement for maintaining the crystal lattice upon dehydration is that the hydrate structure exhibits strong and/or a predominance of host-host interactions. Indeed, the three non-stoichiometric hydrates of the test set show the highest percentage of host-host interactions, i.e., ~90% of the interaction energies for the DDS and indinavir hydrates. This value is lower for brucine (61.5%) but compared to dihydrates showing a stoichiometric behavior, brucine exhibits the most/strongest host-host interactions. The fact that the DDS and indinavir hydrates are isolated-site hydrates and brucine is a channel hydrate highlights that it is not possible to deduce whether a stoichiometic or non-stoichiometric dehydration mechanism occurs from the location of the water molecules in the hydrate structure alone. Though, it is possible to rationalize a non-stoichiometric dehydration behavior from the energy contributions of the intermolecular interactions considering the compound:water ratios. On the other hand, the analysis shows that the moisture- or temperature dependent stability of hydrates cannot be derived from the interaction energy calculations. For example, the 5-flucytosine monohydrate (I) already dehydrates at RH values < 40%, whereas 4-aminoquinaldine monohydrate (Hy1A) dehydrates only at RH values below 10% (RT), but exhibits less energetic contributions from the host-host interactions than the 5-flucytosine monohydrate (I).

In the case of **0.33-Hy** the latter analysis (Figure 10) strongly indicates that water is only weakly bound and rationalizes the facile moisture- and temperature dependent dehydration behavior.

## Discussion

### Molecular level understanding of the dehydration mechanism derived from the hydrate structure

Knowledge of how water vapor is sorbed by a hygroscopic material and how moisture affects the physical and chemical stability of a (pharmaceutical) product is a crucial question in developing drug products or preparations produced from other fine chemicals. Failures and time delays in product developments can be minimized or avoided with knowledge compiled in thorough solid state investigations. Hydrates require a thorough evaluation of their composition and stability under production relevant conditions and additionally the transformation pathways between different solid state forms of a compound, as well as their stability ranges, should be elucidated. This is mandatory to select the ideal solid state form that guarantees an optimal product performance and stability. In general, non-stoichiometric hydrates are undesired solid forms because any change in water vapor pressure of the surrounding medium causes a change in the water content of the substance, which can be critical for weighing and dosing operations and may thus lead to errors in any analyses, which require exact sample amounts. Such variations in the water content are often difficult to avoid as it requires special efforts to precisely control temperature and humidity conditions during processing and storage. Furthermore, the water molecules which have been released from such a hydrate may interact with other excipients in a drug formulation. Gravimetric moisture sorption/desorption studies (Figure 7), combined with environmental PXRD experiments (Supplementary Material) are the preferred analytical techniques for unraveling this non-stoichiometric behavior of a hydrate.

DDS **0.33-Hy** is a prime example for an isolated site hydrate with non-stoichiometric dehydration behavior. The latter behavior may be expected for hydrates where the water is located in open voids such as channels or layers. Thus, this study highlights that the popular structural classification of hydrates into isolated-site hydrates (water molecules are isolated from direct contact), channel hydrates (chains of water molecules) and ion-associated hydrates (metal ions are coordinated with water) cannot be directly related to the dehydration behavior or dehydration mechanism of a hydrate. However, by complementing the structural features with intermolecular energy calculations the observed dehydration behavior can be rationalized. As shown for DDS **0.33-Hy** the water molecules are only weakly bound (Figure 11), allowing a facile water egress/ingress with changing environmental conditions. Furthermore, the energy difference between the isomorphic dehydrate structure and anhydrate polymorphs is small. The lattice energy difference between **Hy**_dehy_ and form **II** was calculated as 1.6 kJ mol^−1^ (PBE-TS, at −273°C) and the transition enthalpy between **Hy**_dehy_ and form **II** determined to be 1.08 ± 0.05 kJ mol^−1^ (experimentally measured at ~100°C). Thus, the calculations rationalize and indicate the non-stoichiometric dehydration mechanism.

### Computational modeling of pharmaceutical hydrates

Modeling and predicting hydrate structures of pharmaceuticals still represent a big challenge in computational chemistry. Numerous potentials have been developed for modeling water (Guillot, 2002), however, there exists no method that can sufficiently model all its abnormalities. In organic hydrates the water molecules may be described as confined at nanoscales, implying frustration in their hydrogen-bonding coordination. Consequently, accurately modeling the water molecules in a hydrate lattice requires modeling efforts which go well beyond the methods applied for modeling the organic solid state (Reilly et al., 2016). In lead optimization is has been demonstrated that incorporating the three-body energy terms, and modeling frustration and frustration-related dielectric responses, significantly improves the results (Fernández, 2016, 2017; Fernandez and Scott, 2017). Considering and modeling the latter can be expected to significantly increase the accuracy of lattice and intermolecular energy calculations of water containing species, albeit at the expense of computational cost.

A major difficulty with using CSP in hydrate solid form screening is the computational expense in time and resources to generate the crystal energy landscape for all possible hydrate stoichiometries (mono-, di-, etc.,). However, solid form modeling at the electronic and atomistic level can provide vital support for unraveling the solid state for a compound which may not be achieved with experiments alone. A CSP study answers the question what types of crystal packings are favorable for a specific molecule, unraveling the compromises between close packing efficiency, conformational preferences and the different types of intermolecular interactions that can lead to feasible structures for a molecule (polymorph) or multi-component system (salt, solvate, hydrate, co-crystal). It should be stressed that CSP aids the interpretation of the experimental data (Price et al., 2014) and can guide experimentalists to find new solid forms (Arlin et al., 2011; Braun et al., 2014b, 2016; Neumann et al., 2015; Srirambhatla et al., 2016).

To significantly reduce the computational cost, and to make the calculations feasible, we did not attempt to computationally screen for different hydrate stoichiometries (Braun et al., 2011b) for the chosen model compound DDS, but used the crystal energy landscape of the 1:1 stoichiometry (monohydrate) as a guidance for hydrate formation. The monohydrate crystal energy landscape (Figure 2) shows only one hydrate structure that is more stable than the non-solvated form **III** and thus indicates hydrate formation.

## Conclusions

4,4′-Diaminodiphenyl sulfone (DDS) forms a non-stoichiometric hydrate, with a water content of 0–0.33 mol of water per mol of DDS. The upper limit of this ratio is obvious from the features of the crystal structure, but it is surprising that the structurally isolated and hydrogen bonded water molecules can easily leave and enter the structure, which is indicated by the continuous change in water content when the hydrate is exposed to different RH values. This observation highlights that it is not advisable to make assumptions about the dehydration behavior based on the location of the water molecules in the structure alone. However, supported by intermolecular energy interaction calculations (host-host, water-host and water-water) and by comparing the lattice energies of the isomorphic dehydrate (hydrate without water) and anhydrate polymorph(s) of the same compound it is possible to rationalize and to potentially predict a non-stoichiometric dehydration behavior. Furthermore, this study shows that even though CSP has been performed with only one hydrate stoichiometry (here monohydrate) the outcome may be sufficient to get insight into the hydrate formation potential of a compound. However, such a limited approach requires a thorough analysis of the computed structures.

In our opinion, a sound understanding of hydrates and their often complex behavior can only be achieved by a full multidisciplinary investigation, including structural, moisture- and temperature dependent studies combined with modeling. Such an understanding may be mandatory to avoid complications during processing, storing and handling of a hydrate.

## Author contributions

DB conceived and designed the research and headed, wrote and revised the manuscript, while UG contributed to the writing and the revision of the article.

### Conflict of interest statement

The authors declare that the research was conducted in the absence of any commercial or financial relationships that could be construed as a potential conflict of interest.
