# Supplementary material for: Supramolecular Organization of Nonstoichiometric Drug Hydrates: Dapsone
Source: Front Chem. 2018 Feb 22;6:31. doi: 10.3389/fchem.2018.00031 (PMC5826966; doi:10.3389/fchem.2018.00031)
Supplement: Supplementary file 1 [file DataSheet1.PDF]

Supplementary Material

Supramolecular Organization of Nonstoichiometric Drug Hydrates: Dapsone

Doris E. Braun* and Ulrich J. Griesser

* **Correspondence:** doris.braun@uibk.ac.at

1 Computationally Generated Low-Energy Monohydrate Structures

Z' = 1 monohydrate structures were randomly generated in following 48 space groups: $P1$, $P\bar{1}$, $P2_1$, $P2_1/c$, $P2_12_12$, $P2_12_12_1$, $Pna2_1$, $Pca2_1$, $Pbca$, $Pbcn$, $C2/c$, Cc, $C2$, Pc, Cm, $P2_1/m$, $C2/m$, $P2/c$, $C222_1$, $Pmn2_1$, $Fdd2$, $Pnna$, $Pccn$, $Pbcm$, $Pnnm$, $Pmmn$, $Pnma$, $P4_1$, $P4_3$, $I\bar{4}$, $P4/n$, $P4_2/n$, $I4/m$, $I4_1/a$, $P41212$, $P4_32_12$, $P3_1$, $P3_2$, $R3$, $P\bar{3}$, $R\bar{3}$, $P3_121$, $P322_1$, $R3$c, $R\bar{3}c$, $P6_1$, $P6_3$, $P6_3/m$.

All calculated structures are available in .res format from the authors on request. The lowest energy PBE-TS structures are given in Supplementary Table 1.

Supplementary Table 1. Computationally generated low-energy dapsone monohydrate structures (PBE-TS).

Str.[a]	Space group	Cell parameters						E_{latt}/ kJ mol^{-1}	PI[b]/ %	H-bonding[c]	Void Space / %
		a/Å	*b*/Å	*c*/Å	*α*/°	*β*/°	*γ*/°				
01_1963	$P2_1$/c	14.029	9.194	9.605	90	100.44	90	–269.54	73.8	DDAA	1.6
02_44	$P2_1$/c	6.111	7.306	28.402	90	92.12	90	–256.67	70.8	DDAA	4.6
03_317	$P2_1$/c	13.375	9.413	10.007	90	94.94	90	–256.14	71.6	DDAA	2.8
04_115	$Pna2_1$	5.610	27.771	7.858	90	90	90	–255.79	74	DDAA	8.5
05_2307	$P2_12_12_1$	5.530	8.182	27.053	90	90	90	–255.50	74	DDAA	7.5
06_246	$P2_12_12_1$	5.653	8.094	26.828	90	90	90	–255.41	73.9	DDAA	7.5
07_29	$Pna2_1$	5.519	27.927	7.913	90	90	90	–254.90	74.2	DDAA	9.0
08_306	$Pccn$	10.694	30.788	7.356	90	90	90	–254.61	74.2	DDA	4.1
09_570	$P2_12_12_1$	5.329	8.772	26.864	90	90	90	–253.60	71.7	DDAA	9.8
10_6827	$P2_12_12_1$	6.005	8.070	25.629	90	90	90	–253.35	72.7	DDAA	0.0
11_6787	Ia	8.250	14.037	11.788	90	101.88	90	–252.26	67.1	DDAA	10.1
12_116	$P2_1$/c	5.471	8.021	27.803	90	93.90	90	–251.89	74.3	DDA	7.6
13_6439	$Pbca$	8.920	9.922	28.595	90	90	90	–251.47	71.3	DDAA	5.5
14_60	$Pbca$	8.897	9.920	28.687	90	90	90	–251.27	71.2	DDAA	5.7
15_18	$C2/c$	10.280	16.574	15.635	90	107.63	90	–251.27	70.8	DDA	5.9
16_322	$P2_1$/c	11.287	14.244	7.667	90	91.79	90	–251.22	73.1	DA	4.4
17_221	$C2/c$	9.888	16.109	16.163	90	96.71	90	–250.77	70.4	DDAA	7.5
18_122	$P2_1$/c	6.084	8.454	24.971	90	94.51	90	–250.48	70.1	DDAA	1.0
19_210	$C2/c$	9.889	16.166	16.138	90	96.93	90	–250.30	70.3	DDAA	7.3
20_8870	$P2_1$/c	10.896	16.659	7.466	90	109.67	90	–250.25	70.5	DDAA	5.8
21_358	$P2_1$	5.784	7.178	15.244	90	96.59	90	–250.03	72	DDAA	8.9
22_362	$P2_1$	5.784	7.163	15.289	90	96.381	90	–249.94	71.9	DDAA	8.7
23_359	$P2_1$	5.765	7.201	15.256	90	95.89	90	–249.81	71.9	DDAA	8.9
24_1127	$Pna2_1$	8.214	14.034	11.581	90	90	90	–249.71	67	DDAA	6.8
25_394	$P2_1$	5.789	7.193	15.220	90	96.32	90	–249.71	71.8	DDAA	8.4
26_386	$P2_1$	5.784	7.223	15.154	90	96.25	90	–249.62	71.9	DDAA	8.6

[a]Structure ID: rank PBE-TS_rank CrystalPredictor. [b]Packing Index calculated using PLATON.(Spek, 2003) [c]water environment type (D – H-bonding donor, A – H-bonding acceptor) (Infantes et al., 2007).

The computational model, PBE-TS, was successful in reproducing the experimental structures (Supplementary Table 2). The computationally generated low energy structures were compared using the Solid Form module of Mercury (Chisholm and Motherwell, 2005) to determine the root mean square deviation of the non-hydrogen atoms in a cluster of 30 molecules ($rmsd_{30}$).

Supplementary Table 2. Quality of representation of the experimental DDS structures.

	Lattice parameters (cell vectors/Å, angles/°)				cell volume (Å^3)	$rmsd_{30}$ (Å)
	a	*b*	*c*	*β*		
Form **III**, DAPSUO05, RT, $P2_12_12_1$	5.758(1)	8.058(1)	25.529(3)	90	1184.49	–
Form **III** PBE-TS, 0 K	5.498	7.975	26.050	90	1142.20	0.335
**0.33-Hy**, ANSFON02, 120 K, *C*2/*c*	48.5832(9)	11.4183(2)	13.0035(2)	92.2789(10)	7207.82	–
**0.33-Hy**, PBE-TS, 0 K	48.1926	11.3940	13.0023	92.100	7134.85	0.052

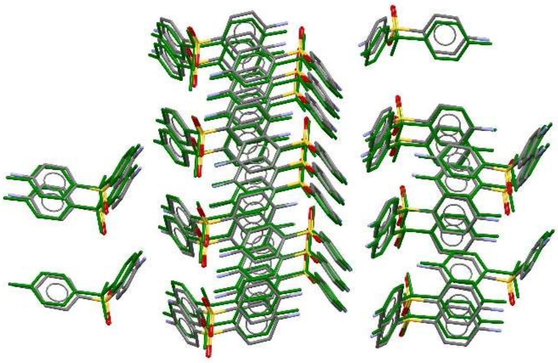

Supplementary Figure 1. Overlay of the 30 molecule cluster of the observed structure of DDS form **III** (coloured by element, DAPSUO05) and calculated PBE-TS structure (green), $rmsd_{30}$=0.335 Å. Hydrogen atoms omitted for clarity.

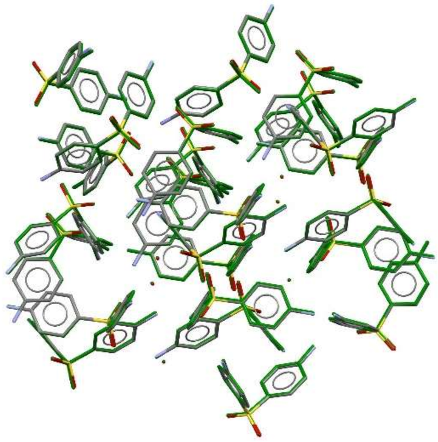

Supplementary Figure 2. Overlay of the 30 molecule cluster of the observed structure of DDS **0.33-Hy** (coloured by element, ANSFON02) and calculated PBE-TS structure (green), $rmsd_{30}$=0.052 Å. Hydrogen atoms omitted for clarity.

2 Energy Framework Calculations

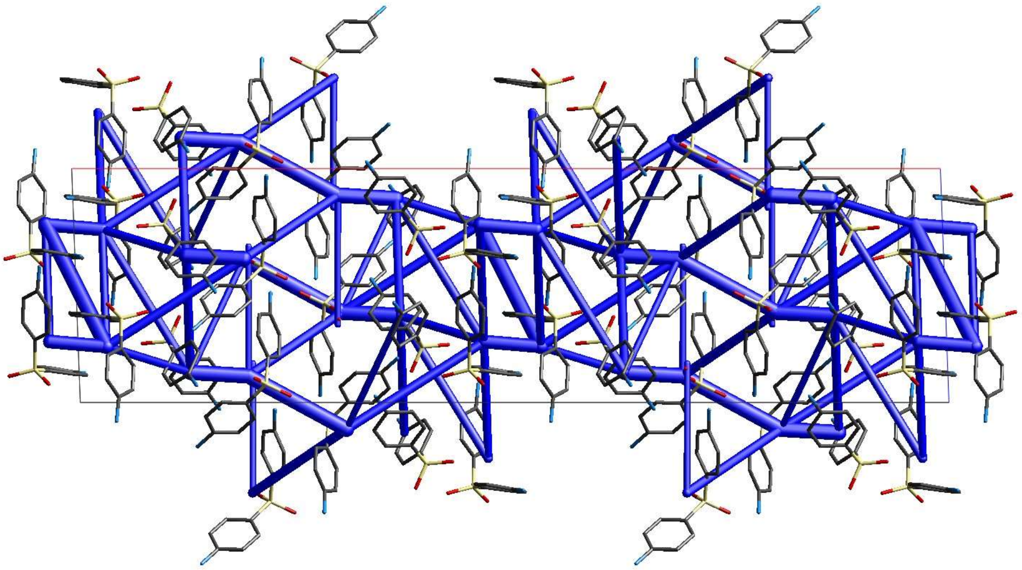

Supplementary Figure 3. Energy frameworks (total energy) for **0.33-Hy**, viewed along the crystallographic *b* axis. The energy scale factor is 80, and interaction energies with magnitudes smaller than 20 kJ mol^{-1} have been omitted.

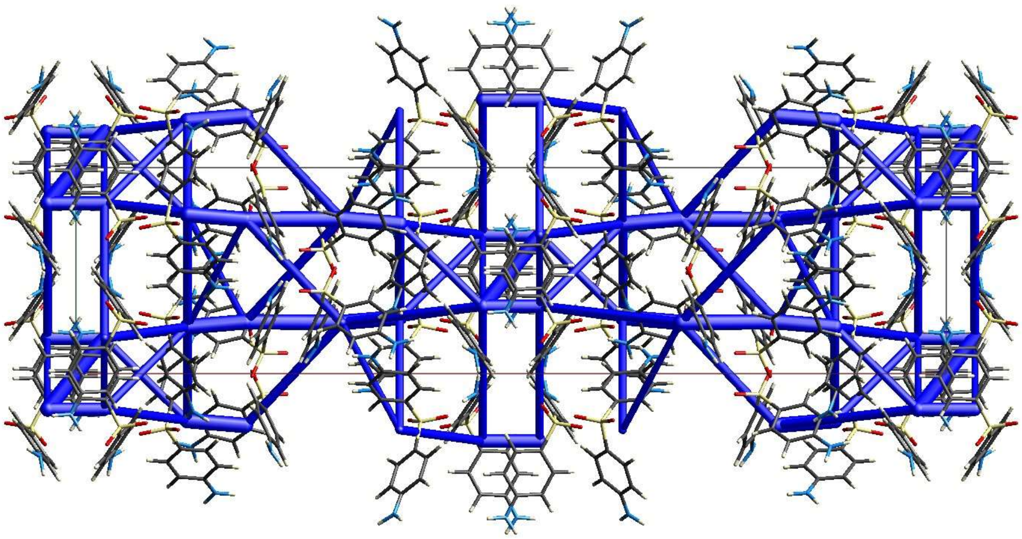

Supplementary Figure 4. Energy frameworks (total energy) for **0.33-Hy**, viewed along the crystallographic *c* axis. The energy scale factor is 80, and interaction energies with magnitudes smaller than 20 kJ mol^{-1} have been omitted.

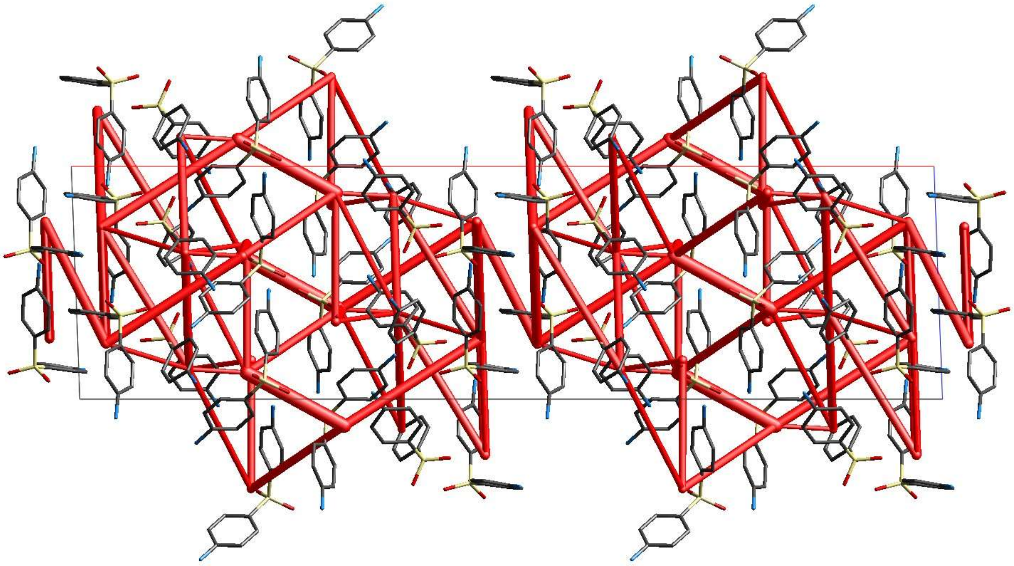

Supplementary Figure 5. Energy frameworks (Coulomb energy) for **0.33-Hy**, viewed along the crystallographic *b* axis. The energy scale factor is 80, and interaction energies with magnitudes smaller than 20 kJ mol^{-1} have been omitted.

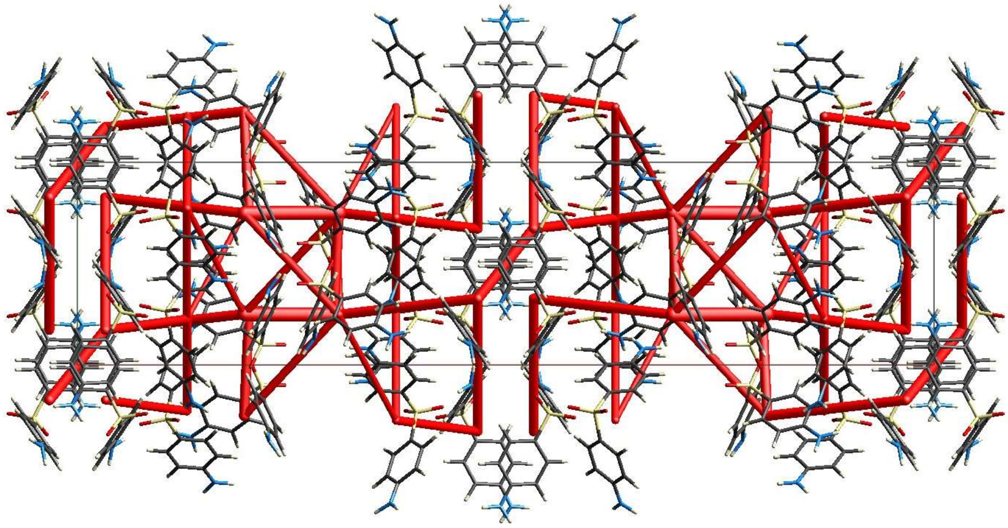

Supplementary Figure 6. Energy frameworks (Coulomb energy) for **0.33-Hy**, viewed along the crystallographic *c* axis. The energy scale factor is 80, and interaction energies with magnitudes smaller than 20 kJ mol^{-1} have been omitted.

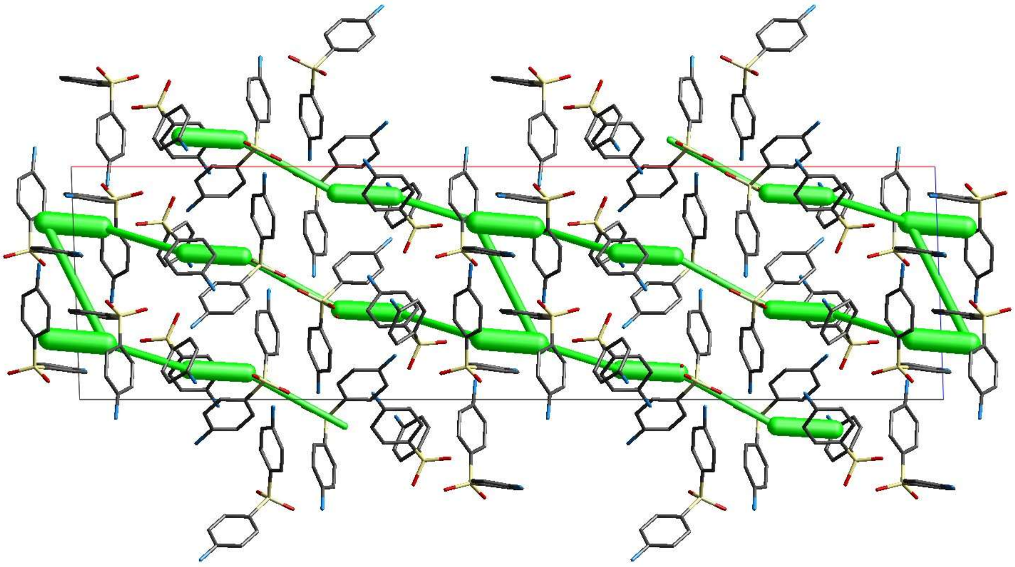

Supplementary Figure 7. Energy frameworks (dispersion energy) for **0.33-Hy**, viewed along the crystallographic *b* axis. The energy scale factor is 80, and interaction energies with magnitudes smaller than 20 kJ mol^{-1} have been omitted.

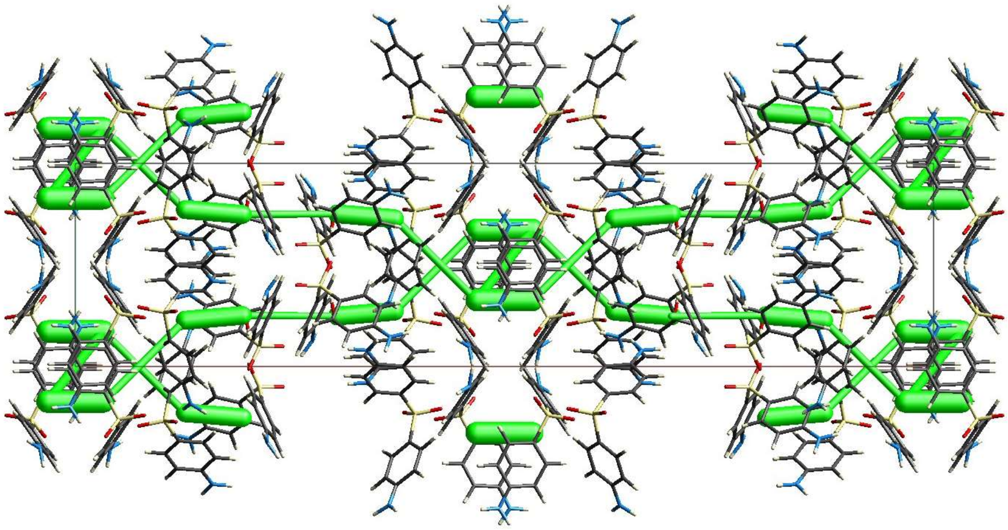

Supplementary Figure 8. Energy frameworks (dispersion energy) for **0.33-Hy**, viewed along the crystallographic *c* axis. The energy scale factor is 80, and interaction energies with magnitudes smaller than 20 kJ mol^{-1} have been omitted.

Supplementary Table 3. Key pair-wise intermolecular interactions present in pharmaceutical/organic hydrates. PBE-TS optimized structures were used.

**No**	**N[b]**	**Inter-action**	**Symmetry operation**	**Centroid distance**	E_E **/ kJ mol^{-1}**	E_P **/ kJ mol^{-1}**	E_D **/ kJ mol^{-1}**	E_R **/ kJ mol^{-1}**	E_{tot} **/ kJ mol^{-1}**
Pyrogallol 0.25-hydrate (ordered structure) - (Braun et al., 2013)									
1	1	host-water	–	4.25	–45.7	–9.2	–8.4	46.9	–33.5
2	1	host-host	–	6.89	–37.3	–8.6	–10.3	36	–32.4
3	1	host-host	–	6.91	–36.8	–8.2	–9.8	35.7	–31.4
4	1	host-water	–	4.37	–32.7	–6.5	–6.5	32.4	–25
5	1	host-host	–	5.93	–24.4	–5.6	–13.6	34.2	–20.7
6	1	host-host	–	5.78	–28.4	–5.4	–12.8	39.8	–20.5
7	1	host-host	–	5.76	–27.5	–5.4	–13.0	39	–20.3
8	2	host-host	x, y, z	3.9	–2	–2.4	–27.8	14.2	–19.4
9	2	host-host	x, y, z	3.9	–2.7	–2.4	–30.7	19.7	–19.2
10	1	host-water	–	3.91	–37.6	–6.3	–8.9	53.5	–19.1
11	1	host-host	–	5.95	–19.4	–5.0	–13.8	28	–18.9
12	2	host-host	x, y, z	3.9	–1.1	–2.3	–27.9	14.5	–18.2
13	2	host-host	x, y, z	3.9	–1.8	–2.2	–30.9	20.9	–17.5
14	1	host-host	–	6.13	–26.6	–4.9	–12.7	41.6	–17.2
15	1	host-host	–	6.11	–26.9	–4.5	–13.3	42.8	–17
16	1	host-host	–	6.45	–22.1	–4.5	–7.9	27.5	–16.6
17	1	host-host	–	6.43	–18.8	–3.6	–7.9	22.1	–15.7
Dapsone 0.33-hydrate (ANSFON02) (Yathirajan, 2004)									
1	1	host-host	–	3.84	–16.0	–5.2	–76.1	53.7	–53.9
2	1	host-host	-x, -y, -z	8.37	–35.4	–6.6	–39.7	38	–53.3
3	1	host-host	-x+1/2, -y+1/2, -z	5.72	–47.1	–15.7	–29.0	54	–53.3
4	1	host-host	-x, y, -z+1/2	3.58	–11.5	–5.4	–89.5	75.9	–47.2
5	1	host-host	–	8.83	–33.6	–9.6	–12.7	32.4	–33.7
6	2	host-host	-x+1/2, y+1/2, -z+1/2	8.31	–28.1	–7.9	–13.6	22.4	–33.5
7	1	host-host	–	9.03	–29.5	–8.4	–10.1	23.5	–31.7
8	2	host-host	x, -y, z+1/2	8.46	–28.7	–7.2	–10.4	21.5	–31.5
9	2	host-host	x, -y, z+1/2	8.84	–27.1	–10.1	–13.7	29.1	–30.1
10	1	host-host	–	6.58	–16.6	–6.4	–32.0	32.9	–29.9
11	2	host-host	x, -y, z+1/2	9.53	–33.1	–10.8	–14.9	42.9	–29.6
12	2	host-host	x, -y, z+1/2	7.86	–15.4	–4.7	–19.4	20.6	–23.9
13	1	host-host	–	9.2	–12.2	–4.5	–16.3	10.7	–23.8
14	1	host-water	–	7.08	–25.9	–6.2	–6.1	23.7	–22.5
15	1	host-water	–	6.62	–16.7	–2.9	–5.7	8.6	–19.5
16	1	host-host	-x+1/2, -y+1/2, -z	11.23	–9.4	–2.2	–11.0	2.8	–19.4
17	1	host-water	–	5.25	–33.8	–7.2	–4.4	41.4	–19.2
18	1	host-host	–	10.37	–20.0	–5.1	–12.6	27.5	–18.9
19	2	host-host	x, -y, z+1/2	8.13	–6.5	–3.8	–9.3	3.9	–15.4

No	N[b]	Inter-action	Symmetry operation	Centroid distance	E_E / kJ mol^{-1}	E_P / kJ mol^{-1}	E_D / kJ mol^{-1}	E_R / kJ mol^{-1}	E_{tot} / kJ mol^{-1}
Neocuproine hemihydrate (TAJRAX) (Britton et al., 1991)									
1	1	host-host	-x+1/2, -y+1/2, -z+1/2	5.33	–10.4	–2.1	–56.6	38.3	–38.2
2	1	host-host	-x, -y+1/2, z	4.21	1.2	–2.9	–62.5	32.7	–35.0
3	1	host-water	–	3.67	–39.5	–9.9	–12.9	43.1	–33.7
4	1	host-water	–	3.67	–39.5	–9.9	–12.9	43.1	–33.7
5	2	host-host	y+1/4, -x+1/4, z+1/4	6.82	–12.6	–4.7	–20.6	11.7	–27.5
6	2	host-host	y+3/4, -x+1/4, -z+1/4	8.35	–4.0	–0.7	–18.5	14	–12.3
7	2	host-host	y+3/4, -x+1/4, -z+1/4	8.72	–1.6	–0.5	–15.5	11.5	–8.4
8	2	host-host	-y+3/4, x+3/4, -z+3/4	8.65	–2.6	–0.6	–12.8	10.1	–8.1
β-Resorcylic acid hemihydrate (QIVTUK01) – (Braun et al., 2011)									
1	1	host-host	-x, -y, -z	7.97	–132.7	–29.8	–13.0	159.3	–75.3
2	1	host-host	-x, -y, -z	7.91	–141.3	–31.5	–13.3	177.8	–74.6
3	1	host-water	–	5.48	–80.3	–18.5	–6.4	100.3	–42.2
4	1	host-host	–	8.58	–49.3	–11.9	–12.2	72.2	–27.0
5	1	host-water	-x, -y, -z	5.98	–8.7	–1.9	–22.4	13.7	–21.7
6	1	host-water	–	4.53	–38.2	–7.4	–6.3	52.4	–19.0
7	1	host-host	–	5.63	–5.9	–1.5	–19.4	9.8	–18.3
8	1	host-water	–	4.89	–2.3	–1.6	–27.4	15.7	–17.7
9	1	host-water	–	5.15	–13.9	–2.4	–3.6	7.2	–15.3
Indinavir monohydrate (DIJWOJ) (Tessadri et al., 2004))									
1	2	host-host	x, y, z	5.7	–99.5	–24.5	–122.4	170.7	–124.6
2	2	host-host	x, y, z	13.44	–58.1	–15.5	–33.3	71.6	–57.6
3	2	host-host	–	11.22	–25.3	–3.8	–75.6	62.2	–57.0
4	1	host-water	–	10.18	–59.5	–13.3	–6.9	74.7	–32.6
5	1	host-water	–	5.64	–32.0	–9.6	–12.6	41.8	–26.0
6	2	host-host	x, y, z	13.86	–7.4	–1.3	–27.7	20.3	–20.4
7	2	host-host	–	12.13	–5.2	–1.0	–26.7	17.9	–18.4
8	2	host-host	–	16.11	–4.6	–2.4	–16.7	14.2	–12.3
9	1	host-water	–	5.95	–4.6	–3.0	–10.7	7.3	–11.9
Ethylmorphine monohydrate (OPOQER) – (Braun et al., 2014)									
1	2	host-host	-x, y+1/2, -z+1/2	8.74	–78.7	–19.5	–30.4	109.9	–56.2
2	1	host-water	–	4.53	–63.7	–12.9	–16.6	64.6	–51.5
3	2	host-host	x+1/2, -y+1/2, -z	8.88	–15.7	–3.2	–45.9	40.4	–33.9
4	2	host-host	x, y, z	6.98	–6.4	–1.6	–32.8	20.7	–23.6
5	2	host-host	-x, y+1/2, -z+1/2	8.48	–8.6	–3.7	–28.9	25.3	–21.4
6	2	host-host	x+1/2, -y+1/2, -z	8.56	–3.6	–0.4	–25.8	14.9	–17.3
7	1	host-water	–	5.1	–10.4	–3.0	–10.2	12.0	–14.7

No	N[b]	Inter-action	Symmetry operation	Centroid distance	E_E / kJ mol^{-1}	E_P / kJ mol^{-1}	E_D / kJ mol^{-1}	E_R / kJ mol^{-1}	E_{tot} / kJ mol^{-1}
Codeine monohydrate (ZZZTZQ02) – (Braun et al., 2014)									
1	2	host-host	-x, y+1/2, -z+1/2	8.48	–68.4	–16.8	–30.2	91.0	–54.8
2	1	host-water	–	4.71	–53.9	–10.1	–14.0	49.0	–46.4
3	2	host-host	x+1/2, -y+1/2, -z	5.53	–18.7	–5.3	–60.1	50.2	–45.0
4	2	host-host	-x, y+1/2, -z+1/2	9.19	–7.6	–1.0	–26.3	23.1	–17.4
5	2	host-host	-x+1/2, -y, z+1/2	8.20	–3.0	–0.4	–23.6	12.5	–16.4
6	1	host-water	–	4.96	–9.1	–2.7	–9.3	8.0	–14.8
7	2	host-host	-x+1/2, -y, z+1/2	10.70	–5.3	–1.5	–8.4	6.9	–9.8
Cytosine monohydrate (CYTOSM13(Lee and Wang, 2010)) – (Braun et al., 2017a)									
1	2	host-host	-x, y+1/2, -z+1/2	5.45	–117.1	–30.0	–16.8	91.7	–104.0
2	1	host-host	-x, -y, -z	4.44	–23.0	–5.2	–21.2	11.2	–39.7
3	1	host-water	–	3.97	–45.9	–11.2	–6.7	48.7	–32.6
4	1	host-water	–	4.53	–43.3	–9.5	–7.9	44.2	–32.4
5	1	host-water	–	4.86	–46.3	–11.0	–3.6	50.1	–29.3
6	1	host-host	-x, -y, -z	6.12	–16.4	–2.8	–4.7	0.4	–23.2
7	2	host-host	x, y, z	7.72	–10.7	–2.3	–2.0	0.4	–14.5
Morphine monohydrate (MORPHMH01(Scheins et al., 2005)) – (Braun et al., 2014)									
1	2	host-host	-x, y+1/2, -z+1/2	7.04	–107.6	–23.2	–45.2	164.9	–68.5
2	1	host-water	–	4.47	–71.9	–12.8	–16.8	95.6	–41.0
3	1	host-water	–	5.54	–64.5	–14.4	–7.8	86.5	–32.3
4	2	host-host	x, y, z	7.31	–4.8	–1.5	–24.4	10.7	–20.9
5	2	host-host	-x+1/2, -y, z+1/2	8.53	–5.8	–1.5	–23.4	16.7	–17.3
6	2	host-host	x+1/2, -y+1/2, -z	8.19	–3.0	–0.5	–18.1	9.5	–13.4
7	2	host-host	-x, y+1/2, -z+1/2	8.92	–11.7	–2.8	–12.2	20.7	–12.3
5-Nitrouracil monohydrate (NURAMH) (Craven, 1967)									
1	1	host-host	-x, -y, -z	6.01	–88.1	–25.0	–17.4	121.7	–51.7
2	1	host-water	–	4.86	–57.4	–11.5	–7.3	38.3	–51.9
3	2	host-host	x, -y+1/2, z+1/2	7.17	–24.2	–6.1	–9.7	18.1	–27.3
4	1	host-water	–	4.8	–24.2	–4.4	–5.7	18.2	–22.5
5	2	host-host	x, y, z	5.21	–2.5	–5.3	–17.3	8.2	–16.6
6	2	host-host	x, y, z	6.17	–6.1	–3.7	–9.6	2.7	–15.8
7	1	host-host	-x, -y, -z	7.20	–5.8	–0.8	–8.3	3.0	–12.1
8	1	host-water	–	5.8	–23.9	–4.9	–4.0	33.9	–11.5

No	N[b]	Inter-action	Symmetry operation	Centroid distance	E_E / kJ mol^{-1}	E_P / kJ mol^{-1}	E_D / kJ mol^{-1}	E_R / kJ mol^{-1}	E_{tot} / kJ mol^{-1}
Orotic acid monohydrate OROTAC01(Portalone, 2008) – (Braun et al., 2016a)									
1	1	host-water	–	5.43	–105.6	–26.0	–5.8	130.5	–55.3
2	1	host-host	-x, -y, -z	7.17	–92.8	–23.5	–16.1	123	–53.6
3	1	host-host	-x, -y, -z	6.06	–79.8	–17.9	–15.9	98.2	–50.8
4	1	host-host	-x, -y, -z	4.34	–14.9	–3.5	–29.0	21.7	–30.2
5	1	host-water	–	5.07	–49.5	–9.4	–4.6	56.5	–28.5
6	1	host-host	-x, -y, -z	6.99	–17.4	–2.2	–9.0	6.1	–24.2
7	1	host-host	-x, -y, -z	5.53	–9.3	–1.0	–13.4	4.7	–19.4
8	1	host-host	-x, -y, -z	6.32	–9.3	–2.7	–9.9	5.3	–17.2
5-Flucytosine monohydrate I (BIRMEU02(Hulme and Tocher, 2006) – (Braun et al., 2017a)									
1	1	host-host	–	5.72	–136.6	–34.3	–19.1	132.2	–104.8
2	1	host-host	–	5.76	–128.6	–31.5	–18.2	116.9	–103.0
3	1	host-water	–	4.88	–35.2	–7.4	–4.6	25.4	–31.1
4	2	water-water	–	2.72	–50.3	–10.1	–4.2	55.4	–30.1
5	2	host-host	-x, y+1/2, -z+1/2	5.82	–17.0	–3.0	–8.6	1.8	–26.6
6	2	host-host	-x, y+1/2, -z+1/2	5.60	–15.7	–3.2	–11.4	4.4	–26.2
7	1	host-water	–	4.92	–42.7	–9.8	–4.4	49.5	–25.7
8	1	host-host	–	5.22	–43.5	–10.2	–3.8	51.6	–25.0
9	2	water-water	–	2.77	–40.0	–9.1	–4.2	46.0	–24.2
10	1	host-water	–	4.87	–16.8	–5.0	–5.4	16.9	–15.7
1,10-Phenanthroline monohydrate (ZZZAMS05(Bolte, 2008)) – (Braun et al., 2017b)									
1	3	host-water (strongest)	–	4.22	-43.8	-12.2	-10	51.1	-32.5
2	3	host-host (strongest)	–	5.34	-15.6	-2.7	-36.2	37.5	-26.9
3	3	host-host (strongest)	–	5.85	-13.6	-3.4	-27.1	24.3	-25.5
4	3	host-water (strongest + weakest)	–	5.73<br>5.33	-15.8<br>-10.5	-3.4<br>-1.6	-28.9<br>-4.9	33.2<br>4.3	-23.8<br>-13.8
5	3	host-host (strongest)	–	6.31	-15.6	-4.1	-21.5	23.9	-23.6
6	3	water-water (strongest)	–	2.95	-28.6	-5.5	-2.8	22.1	-23.2
4-Aminoquinaldine monohydrate A (LOBSOL(Tai et al., 2008)) – (Braun et al., 2016b)									
1	2	host-host	x, y, z	4.57	–0.5	–3.5	–46.0	30.5	–24.3
2	1	host-water	–	4.16	–80.1	–18.6	–13.4	114.5	–39.4
3	1	host-water	–	5.42	–40.7	–8.7	–5.0	41.0	–28.5
4	2	water-water	x+1/2, -y+1/2, z	2.7	–50.9	–11.2	–3.8	63.4	–26.3
5	2	host-host	x+1/2, -y+1/2, z	6.33	–13.1	–4.0	–23.8	29.7	–19.2
6	2	host-host	-x, -y, z+1/2	7.53	–6.8	–0.9	–13.0	11.2	–12.3

No	N[b]	Inter-action	Symmetry operation	Centroid distance	E_E / kJ mol^{-1}	E_P / kJ mol^{-1}	E_D / kJ mol^{-1}	E_R / kJ mol^{-1}	E_{tot} / kJ mol^{-1}
Orcinol monohydrate (EWAMIZ) (Mukherjee et al., 2011)									
1	1	host-water	–	4.7	–58.8	–13.7	–6.6	61.9	–39.8
2	1	host-water	–	4.57	–62.7	–14.6	–7.5	73.7	–38.1
3	1	host-water	–	4.67	–44.5	–8.1	–8.0	60.3	–22.7
4	2	host-host	x, -y+1/2, z+1/2	4.48	–4.9	–1.9	–29.4	21.2	–19.1
5	1	host-host	-x, -y, -z	6.84	–8.4	–1.1	–7.8	2.2	–15.1
6	1	host-water	–	4.18	–39.0	–7.4	–7.9	63	–14.6
7	1	host-host	-x, -y, -z	5.84	–4.4	–1.2	–11.9	5.9	–12.3
Brucine dihydrate (CIKDOQ(Smith et al., 2007) – (Braun and Griesser, 2016)									
1	2	host-host	-x, y+1/2, -z	7.55	–22.1	–8.8	–72.3	52.4	–60.5
2	2	host-host	x, y, z	7.41	–13.6	–2.9	–64.8	40.5	–47.9
3	1	host-host	–	7.54	–23	–4.2	–57.5	50.7	–46.1
4	1	host-water	–	5.44	–63.1	–15.4	–14.5	84.4	–38.7
5	1	host-host	–	9.73	–16.8	–5.1	–35.2	28.8	–34.4
6	1	water-water	–	2.69	–53.8	–10.8	–4.4	59.5	–31.9
7	1	water-water	–	2.69	–56.3	–11.8	–4.4	65.6	–31.5
8	1	host-water	–	5.75	–29.7	–6.1	–6.8	19.2	–30.0
9	1	water-water	–	2.75	–47.3	–9.7	–3.9	50.2	–29.6
10	1	water-water	–	2.73	–48	–9.4	–3.6	52.1	–28.7
11	1	host-host	–	9.29	–10.8	–2.4	–33.3	23.7	–27.5
12	1	host-water	–	6.3	–42.2	–9.7	–5.5	50.2	–25.6
13	1	host-host	–	9.56	–9.2	–4.8	–30.9	28.2	–22.8
14	1	host-host	–	10.41	–5.8	–0.9	–24.4	12.1	–20.6
15	2	host-host	-x, y+1/2, -z	9.18	–3.5	–1	–22.8	9.8	–18.3
16	1	host-water	–	5.05	–13.1	–4	–10.4	14.2	–17.0
Propylgallate dihydrate, Hy A (FACVAH) (Okabe and Kyoyama, 2002)									
1	1	host-host	-x, -y, -z	4.45	–24.3	–3.7	–65.7	53.5	–52.6
2	1	host-water	-x, -y, -z	3.52	–12.9	–4.3	–62.2	44.3	–43.7
3	1	host-water	–	6.17	–62.2	–14.8	–5.8	69.9	–38.6
4	2	host-water	x, y, z	7.41	–35.9	–6.6	–11.3	24.7	–37.4
5	1	host-water	–	6.29	–52.7	–11.9	–7.2	58.4	–34.7
6	1	host-water	–	5.28	–45.7	–10.0	–9.0	50.6	–32.3
7	1	water-water	–	2.71	–49.9	–10.2	–3.4	58.4	–27.2
8	1	host-water	–	4.63	–25	–4.9	–5.7	21.1	–22.1
9	1	host-water	–	5.51	–34.3	–6.4	–6.2	44.1	–19.2
10	1	host-host	-x, -y, -z	7.58	–6.5	–1.5	–10.5	5.7	–13.6

No	N[b]	Inter-action	Symmetry operation	Centroid distance	E_E / kJ mol^{-1}	E_P / kJ mol^{-1}	E_D / kJ mol^{-1}	E_R / kJ mol^{-1}	E_{tot} / kJ mol^{-1}
Barbituric acid dihydrate (BARBAD15(Nichol Gary and Clegg, 2005) – (Zencirci et al., 2009)									
1	2	host-host	x+1/2, -y+1/2, -z	6.33	–53.1	–10.9	–11.2	62.8	–35.1
2	1	host-water	–	4.05	–72.5	–15.7	–6.8	80.5	–44.5
3	1	water-water	–	2.7	–52.8	–11.3	–3.3	65.6	–26.6
4	1	water-water	–	5.21	–33.2	–5.8	–3.8	36.6	–20.0
5	1	host-water	–	3.07	–10.3	–2.8	–10.1	7	–17.3
6	1	host-water	–	4.93	–35.9	–8.1	–5.3	52.8	–15.9
7	2	host-host	-x, y+1/2, -z+1/2	5.61	–7.9	–1.3	–10.0	4.5	–15.3
8	2	host-host	-x, y+1/2, -z+1/2	6.15	–7.1	–1.1	–7.4	2.4	–13.3
Neocuproine dihydrate (SAZXAS01) (Koeroglu et al., 2005)									
1	1	host-water	–	3.69	–40.8	–9.6	–12.2	36.7	–38.2
2	1	host-water	–	3.66	–40.2	–10.5	–12.3	40.5	–36.0
3	1	host-water	-x, -y, -z	6.20	–10.2	–1.6	–49.0	35.8	–32.6
4	1	host-host	-x, -y, -z	6.48	–9.7	–1.7	–48.0	34.8	–31.8
5	2	host-host	-x, y+1/2, -z+1/2	6.51	–10.1	–1.7	–48.4	36.2	–31.8
6	1	water-water	–	2.73	–50.6	–10.7	–3.9	55.5	–30.5
7	1	water-water	–	2.79	–38.4	–7.6	–3.4	39.4	–24.9
Phloroglucinol dihydratre (ordered structure) – (Braun et al., 2012)									
1	1	host-water	–	4.83	–60.3	–13.5	–5.7	60.8	–41.1
2	1	host-water	–	4.6	–55.4	–12.9	–7.4	58.7	–38.3
3	1	host-water	–	4.62	–56.2	–13.5	–7.2	63.6	–36.4
4	1	water-water	–	2.7	–53.9	–11.2	–3.9	63.5	–29.5
5	2	host-host	-x, y+1/2, -z+1/2	5.62	–12.4	–2.1	–11.9	4.2	–22.3
6	1	host-water	–	4.62	–39.1	–7.3	–8.6	56.3	–19.5
7	1	host-water	–	4.46	–33.5	–6.4	–9.5	47.9	–18.8
8	1	host-water	–	3.88	–11.9	–2.2	–9.1	10.5	–15.6
9	2	host-host	-x, y+1/2, -z+1/2	4.59	–2.7	–2.5	–25.3	18.4	–15.4

[a]Electrostatic (E_E), polarization (E_P), dispersion (E_D) and exchange-repulsion energy (E_R). $E_{tot} = k_E E_E + k_P E_P + k_D E_D + k_R E_R$, with k being scale factors (Mackenzie et al., 2017). [b]Times the interaction is present.

Supplementary Table 4. Intermolecular interaction energy calculations ($E_{cluster}$) grouped into contributions arising from host-host ($E_{host-host}$), host-water ($E_{host-water}$) and water-water ($E_{water-water}$) interactions.[a]

Hydrate (Host:Water)	$E_{cluster}$ / kJ mol^{-1}	$E_{host-host}$ / kJ mol^{-1}	$E_{host-water}$ / kJ mol^{-1}	$E_{water-water}$ / kJ mol^{-1}	Ratio[b] host/water	Molar Volume (Host+Water) / Å^3	% Water of Molar Volume / %
Pyrogallol (4:1)	–281.85	–234.95	–47.6	0.7	5.01	451.73	4.28
Dapsone (3:≤1)	–428.60	–387.05	–41.55	0.00	9.32	647.71	2.98
Neocuproine (2:1)	–143.30	–103.90	–39.40	0.00	2.64	413.01	4.68
beta Resorcylic Acid (2:1)	–202.40	–129.20	–70.75	–2.45	1.77	273.49	7.07
Indinavir (1: ≤1)	–333.55	–296.00	–37.55	0.00	7.88	609.46	3.17
Ethylmorphine (1:1)	–201.20	–162.80	–38.40	0.00	4.24	310.34	6.23
Codeine (1:1)	–189.70	–152.70	–37.00	0.00	4.13	293.54	6.59
Morphine (1:1)	–198.10	–148.70	–46.30	–3.10	3.01	276.01	7.00
Cytosine (1:1)	–185.50	–139.15	–52.85	6.50	3.00	114.47	16.89
5-Nitrouracil (1:1)	–136.10	–91.10	–44.40	–0.60	2.02	134.65	14.36
Orotic Acid (1:1)	–168.00	–107.90	–57.75	–2.35	1.80	138.31	13.98
5-Flucytosine (1:1)	–138.50	–85.00	–21.95	–31.55	1.59	119.41	16.19
o-Phenanthroline (1:1)	–106.47	–55.27	–28.50	–22.70	1.08	183.04	10.56
4-Aminoquinaldine (1:1)	–136.90	–70.20	–40.40	–26.30	1.05	171.06	11.30
Orcinol (1:1)	–118.95	–53.50	–59.65	–5.80	0.82	135.97	14.22
Brucine (1: ≤2)	–194.48	–119.45	–41.30	–33.73	1.59	391.15	9.88
Propylgallat (1:2)	–215.05	–112.15	–89.40	–13.50	1.09	224.89	17.19
Barbituric Acid (1:2)	–146.10	–67.00	–54.90	–24.20	0.85	139.01	27.81
Neocuproine (1:2)	–173.75	–71.60	–71.00	–31.15	0.70	235.5	16.42
Phloroglucinol (1:2)	–161.60	–40.90	–107.55	–13.15	0.34	146.76	26.34

[a]Pair-wise interaction energies divided by 2. [b]$E_{host-host}$/($E_{host-water}$ + $E_{water-water}$).

3 Dispersion Corrected Density Functional Calculations (PBE-TS)

Supplementary Table 5. PBE-TS calculations on **0.33-Hy** and lower hydrates thereof.

**Structure ID**	**Water content** mol H_2O/mol DDS	E_{CASTEP}[a] / kJ mol^{-1}	E_{latt} / kJ mol^{-1}	**Contribution of water to** E_{latt}[b] / kJ mol^{-1}
**0.33-Hy**	0.33	–376663.9157	–222.95	–25.69
W1	0.25	–372888.1934	–216.98	–19.73
W12	0.17	–369112.0129	–210.56	–13.31
W13	0.17	–369112.197	–210.75	–13.49
W14	0.17	–369111.7146	–210.27	–13.01
W123	0.08	–365335.6176	–203.93	–6.67
**Hy$_{dehy}$**	0.00	–361559.1846	–197.26	0.00

[a]CASTEP energies calculated using the settings given in section 2.1. of the manuscript.
[b]E_{latt}(structure ID) – E_{latt}(**Hy$_{dehy}$**).

4 Water Activity Measurements (Slurry Method)

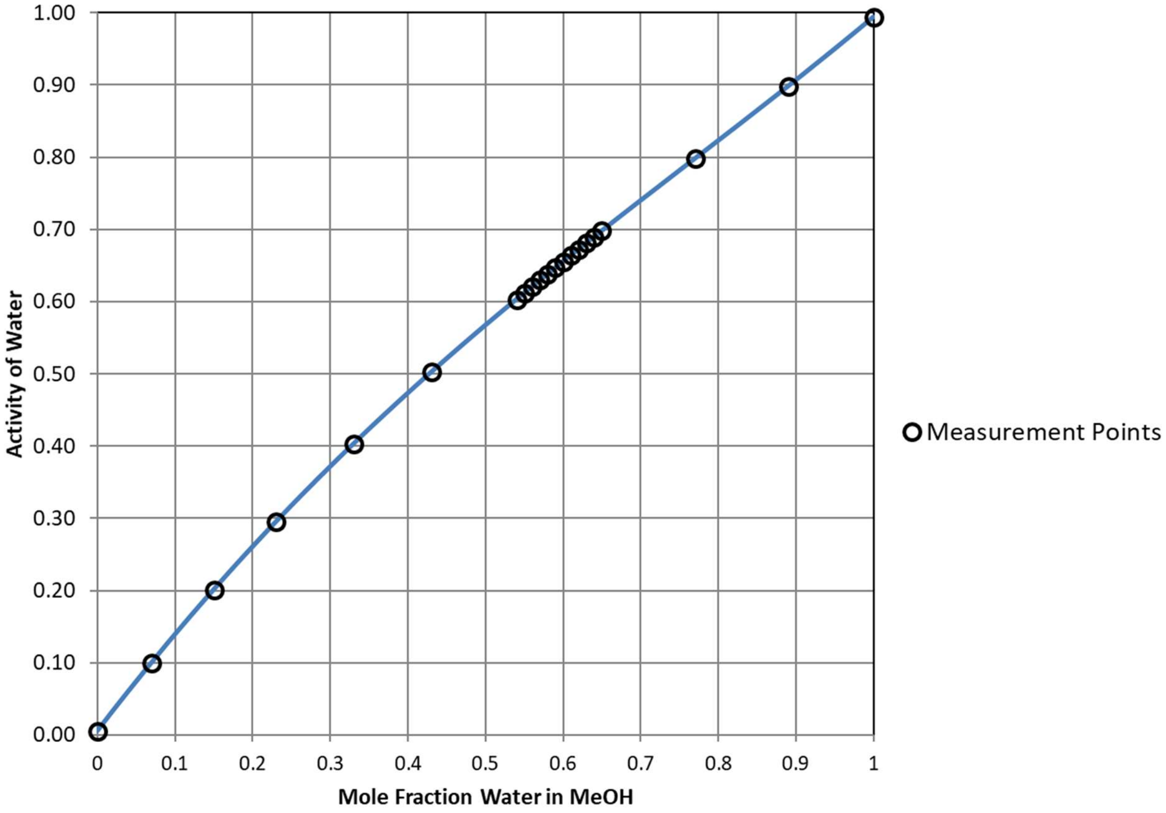

Supplementary Figure 9. Plot of the water activity *vs.* the mole fraction of water in methanol/water mixtures as 25 °C. Data from (Zhu et al., 1996).

5 Variable RH Powder X-ray Diffraction Experiments: Rietveld Refinement

Supplementary Table 6. Refined unit cell parameters, volume and water occupancy dependence on RH for DDS **0.33-Hy** at 25 °C. Powder patterns shown in Supplementary Figure 10 and Supplementary Figure 11.

RH /%	a / Å	b / Å	c / Å	β / °	Vol / $Å^3$	R_{wp} / %	R_{exp} / %	R_p / %	GOF	Occ. (H_2O)
90	48.9832(10)	11.4232(2)	13.1052(3)	92.641(1)	7325.16(30)	6.07	3.04	4.64	2.00	0.888
80	48.9791(10)	11.4227(2)	13.1043(3)	92.641(1)	7323.76(30)	6.09	3.01	4.67	2.03	0.843
70	48.9775(10)	11.4228(2)	13.1029(3)	92.641(1)	7322.79(30)	6.05	3.01	4.65	2.01	0.806
60	48.9752(11)	11.4223(2)	13.1015(3)	92.646(1)	7321.24(31)	6.18	3.01	4.76	2.05	0.773
50	48.9715(11)	11.4219(2)	13.0994(3)	92.650(1)	7319.29(31)	6.13	3.01	4.68	2.03	0.739
40	48.9655(11)	11.4214(2)	13.0961(3)	92.651(1)	7316.20(31)	6.07	3.02	4.63	2.01	0.693
30	48.9581(11)	11.4206(2)	13.0919(3)	92.656(1)	7312.23(31)	6.10	3.02	4.68	2.02	0.629
20	48.9455(11)	11.4192(2)	13.0859(3)	92.663(1)	7306.08(31)	6.08	3.01	4.68	2.02	0.532
10	48.9302(11)	11.4168(3)	13.0780(3)	92.671(1)	7297.75(32)	6.27	3.00	4.78	2.09	0.367
1	48.8904(12)	11.4071(3)	13.0628(3)	92.694(1)	7277.03(33)	6.65	2.99	5.09	2.22	0.005
10	48.9295(11)	11.4165(3)	13.0778(3)	92.673(1)	7297.35(32)	6.23	3.01	4.75	2.07	0.367
20	48.9464(11)	11.4189(3)	13.0857(3)	92.663(1)	7305.92(31)	6.12	3.01	4.71	2.04	0.532
30	48.9578(11)	11.4206(2)	13.0914(3)	92.656(1)	7311.87(31)	6.04	3.03	4.63	1.99	0.629
40	48.9639(11)	11.4211(3)	13.0955(3)	92.652(1)	7315.43(31)	6.14	3.03	4.67	2.03	0.693
50	48.9699(11)	11.4218(2)	13.0984(3)	92.649(1)	7318.46(31)	6.09	3.03	4.70	2.01	0.739
60	48.9722(10)	11.4218(2)	13.1005(3)	92.645(1)	7319.93(30)	6.04	3.03	4.60	2.00	0.773
70	48.9754(10)	11.4220(2)	13.1025(3)	92.642(1)	7321.70(30)	6.08	3.03	4.63	2.01	0.806
80	48.9773(10)	11.4220(2)	13.1035(3)	92.638(1)	7322.62(30)	6.09	3.03	4.68	2.01	0.843
90	48.9832(10)	11.4232(2)	13.1052(3)	92.641(1)	7325.16(30)	6.07	3.04	4.64	2.00	0.888

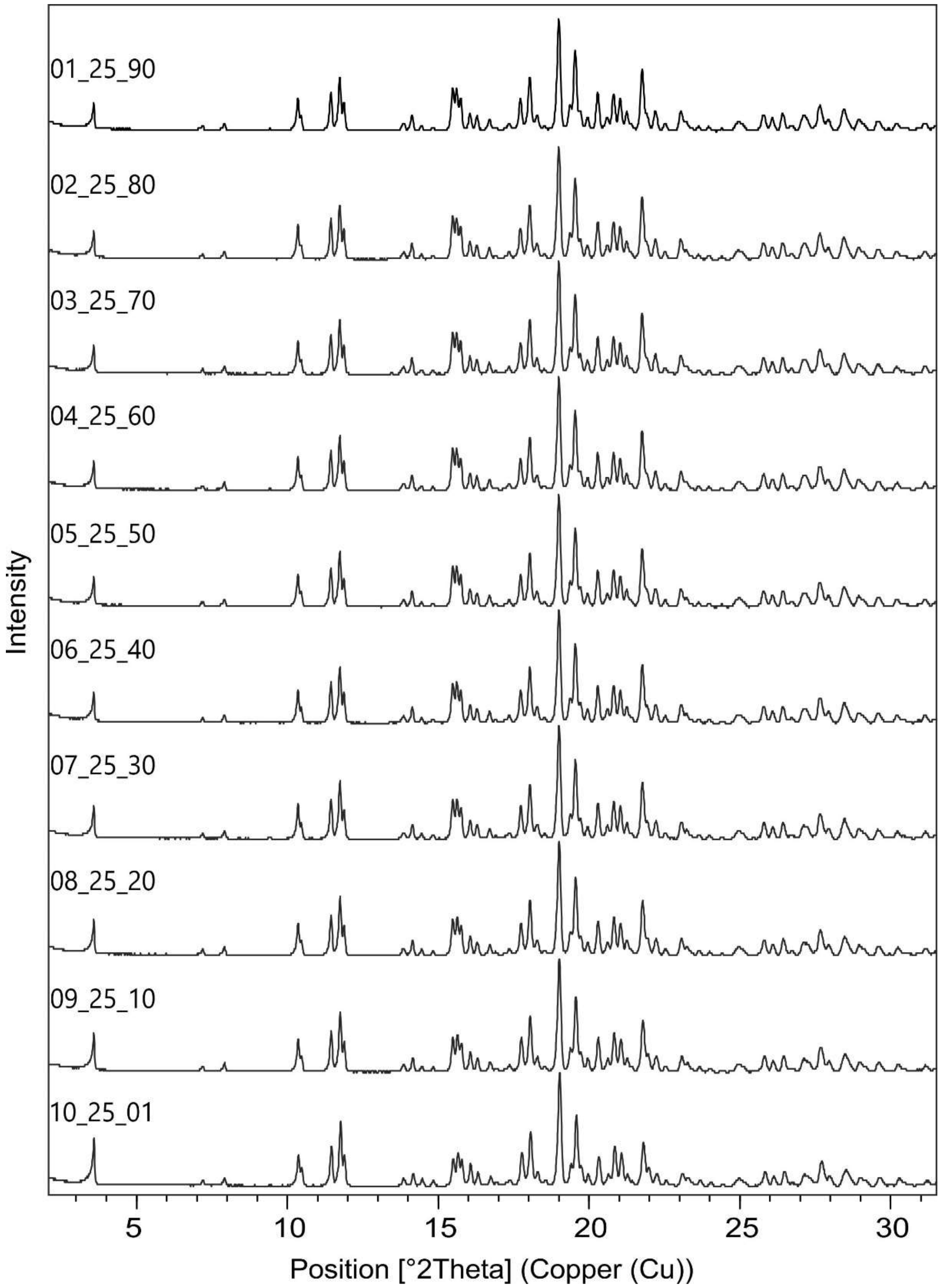

Supplementary Figure 10. Moisture dependent PXRD measurements of DDS **0.33-Hy** – desorption. Labelling: ID_temperature (°C)_RH (%).

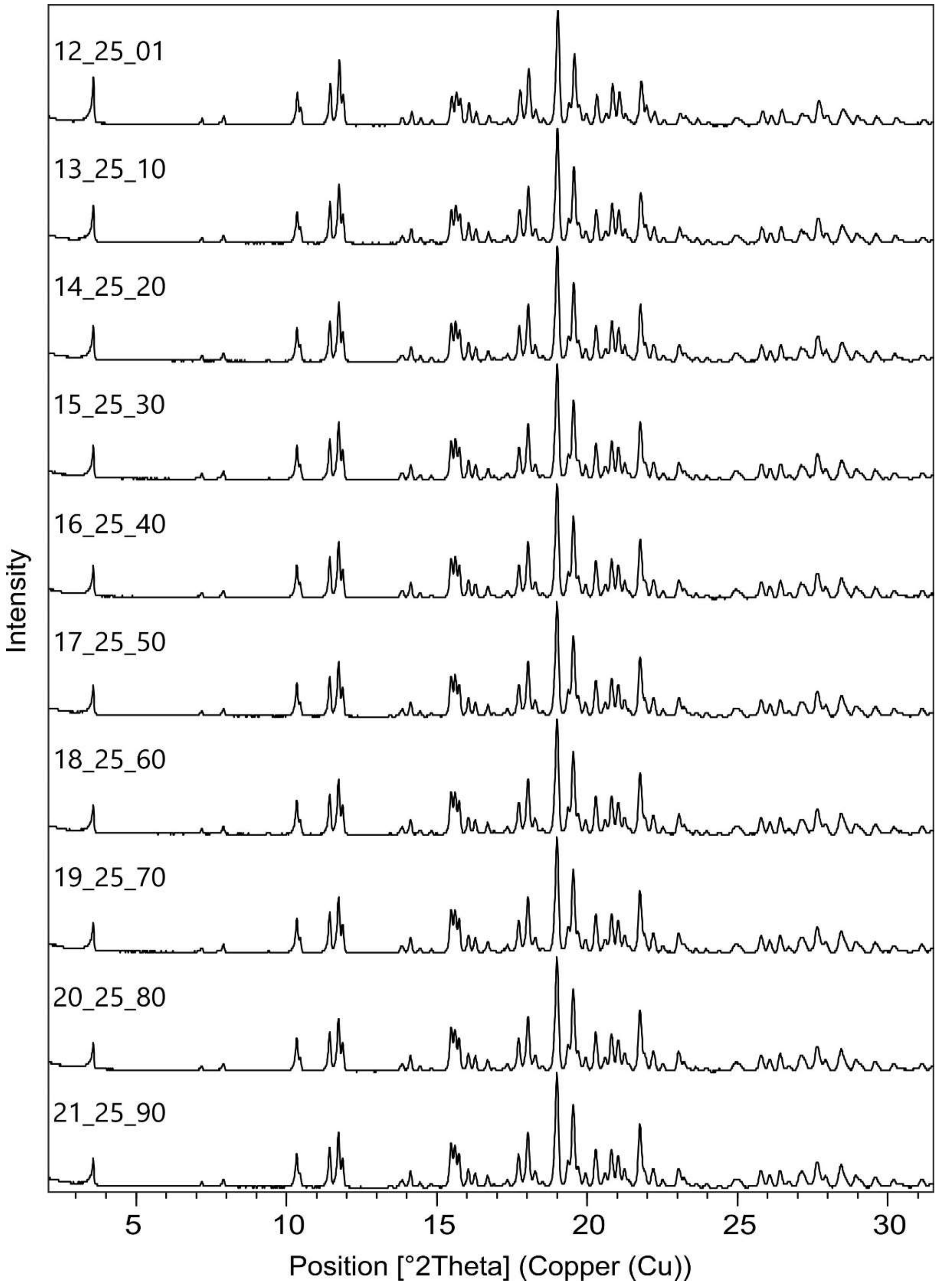

Supplementary Figure 11. Moisture dependent PXRD measurements of DDS **0.33-Hy** – sorption. Labelling: ID_temperature (°C)_RH (%).

6 References

Bolte, M. (2008), *CSD Communication.*

Braun, D.E., Bhardwaj, R.M., Arlin, J.B., Florence, A.J., Kahlenberg, V., Griesser, U.J., Tocher, D.A., and Price, S.L. (2013). Absorbing a Little Water: The Structural, Thermodynamic, and Kinetic Relationship between Pyrogallol and Its Tetarto-Hydrate. *Cryst. Growth Des* 13**,** 4071-4083.

Braun, D.E., Gelbrich, T., Kahlenberg, V., and Griesser, U.J. (2014). Insights into Hydrate Formation and Stability of Morphinanes from a Combination of Experimental and Computational Approaches. *Molecular Pharmaceutics* 11**,** 3145-3163.

Braun, D.E., and Griesser, U.J. (2016). Stoichiometric and Non-Stoichiometric Hydration of Brucine. *Cryst. Growth Des* 16**,** 6111-6121.

Braun, D.E., Kahlenberg, V., and Griesser, U.J. (2017a). Experimental and Computational Hydrate Screening: Cytosine, 5-Flucytosine, and Their Solid Solution. *Cryst. Growth Des.* 17**,** 4347-4364.

Braun, D.E., Karamertzanis, P.G., Arlin, J.B., Florence, A.J., Kahlenberg, V., Tocher, D.A., Griesser, U.J., and Price, S.L. (2011). Solid-State Forms of beta -Resorcylic Acid: How Exhaustive Should a Polymorph Screen Be? *Cryst. Growth Des* 11**,** 210-220.

Braun, D.E., Nartowski, K.P., Khimyak, Y.Z., Morris, K.R., Byrn, S.R., and Griesser, U.J. (2016a). Structural Properties, Order-Disorder Phenomena and Phase Stability of Orotic Acid Crystal Forms. *Mol. Pharm* 13**,** 1012-1029.

Braun, D.E., Oberacher, H., Arnhard, K., Orlova, M., and Griesser, U.J. (2016b). 4-Aminoquinaldine monohydrate polymorphism: prediction and impurity aided discovery of a difficult to access stable form. *CrystEngComm* 18**,** 4053-4067.

Braun, D.E., Schneeberger, A., and Griesser, U.J. (2017b). Understanding the role of water in 1,10-phenanthroline monohydrate. *CrystEngComm***,** Ahead of Print.

Braun, D.E., Tocher, D.A., Price, S.L., and Griesser, U.J. (2012). The Complexity of Hydration of Phloroglucinol: A Comprehensive Structural and Thermodynamic Characterization. *J. Phys. Chem. B* 116**,** 3961-3972.

Britton, D., Thompson, L.C., and Holz, R.C. (1991). Structure of 2,9-dimethyl-1,10-phenanthroline hemihydrate. *Acta Crystallogr., Sect. C Cryst. Struct. Commun.* C47**,** 1101-1103.

Chisholm, J.A., and Motherwell, S. (2005). COMPACK: a program for identifying crystal structure similarity using distances. *Journal of Applied Crystallography* 38**,** 228-231.

Craven, B.M. (1967). The crystal structure of 5-nitrouracil monohydrate. *Acta Crystallographica* 23**,** 376-383.

Hulme, A.T., and Tocher, D.A. (2006). The Discovery of New Crystal Forms of 5-Fluorocytosine Consistent with the Results of Computational Crystal Structure Prediction. *Crystal Growth & Design* 6**,** 481-487.

Infantes, L., Fabian, L., and Motherwell, W.D.S. (2007). Organic crystal hydrates: what are the important factors for formation. *CrystEngComm* 9**,** 65-71.

Koeroglu, A., Bulut, A., Ucar, I., Nichol, G.S., Harrington, R.W., and Clegg, W. (2005). A second monoclinic polymorph of 2,9-dimethyl-1,10-phenanthroline dihydrate. *Acta Crystallogr., Sect. E Struct. Rep. Online* 61, o3723-o3725.

Lee, T., and Wang, P.Y. (2010). Screening, Manufacturing, Photoluminescence, and Molecular Recognition of Co-Crystals: Cytosine with Dicarboxylic Acids. *Cryst. Growth Des* 10, 1419-1434.

Mackenzie, C.F., Spackman, P.R., Jayatilaka, D., and Spackman, M.A. (2017). CrystalExplorer model energies and energy frameworks: extension to metal coordination compounds, organic salts, solvates and open-shell systems. *IUCrJ* 4.

Mukherjee, A., Grobelny, P., Thakur, T.S., and Desiraju, G.R. (2011). Polymorphs, Pseudopolymorphs, and Co-Crystals of Orcinol: Exploring the Structural Landscape with High Throughput Crystallography. *Cryst. Growth Des.* 11, 2637-2653.

Nichol Gary, S., and Clegg, W. (2005). A variable-temperature study of a phase transition in barbituric acid dihydrate. *Acta Crystallogr B* 61, 464-472.

Okabe, N., and Kyoyama, H. (2002). Propyl gallate dihydrate. *Acta Crystallogr., Sect. E Struct. Rep. Online* 58, o245-o247.

Portalone, G. (2008). Redetermination of orotic acid monohydrate. *Acta Crystallogr. , Sect. E: Struct. Rep. Online* 64, o656, o656/651-o656, o656/656.

Scheins, S., Messerschmidt, M., and Luger, P. (2005). Submolecular partitioning of morphine hydrate based on its experimental charge density at 25 K. *Acta Crystallogr B* 61, 443-448.

Smith, G., Wermuth, U.D., and White, J.M. (2007). Pseudopolymorphism in brucine: brucine-water (1/2), the third crystal hydrate of brucine. *Acta Crystallogr. C* 63, o489-o492.

Spek, A.L. (2003). "PLATON, A Multipurpose Crystallographic Tool". (Utrecht, The Netherlands: Utrecht University).

Tai, X.S., Xu, J., Feng, Y.M., and Liang, Z.P. (2008). 4-Amino-2-methylquinoline monohydrate. *Acta Crystallogr. , Sect. E: Struct. Rep. Online* 64, o1026, o1026/1021-o1026, o1026/1026.

Tessadri, R., Griesser, U.J., Prillinger, M., and Wurst, K. (2004). Indinavir: a rare urinary stone in HIV-1-infected patients - occurrence and crystal properties. *Mater. Sci. Forum* 443-444, 407-410.

Yathirajan, H.S., Nagaraja, P., Nagaraj, B., Bhaskar, B.L., Lynch, D.E. (2004) *CSD Communication*.

Zencirci, N., Gstrein, E., Langes, C., and Griesser, U.J. (2009). Temperature- and moisture-dependent phase changes in crystal forms of barbituric acid. *Thermochimica Acta* 485, 33-42.

Zhu, H., Yuen, C., and Grant, D.J.W. (1996). Influence of water activity in organic solvent + water mixtures on the nature of the crystallizing drug phase. 1. Theophylline. *Int. J. Pharm* 135, 151-160.
